# Cell-type resolved transcriptional network analysis of *in vivo* cellular senescence following injury

**DOI:** 10.1371/journal.pcbi.1014429

**Published:** 2026-06-22

**Authors:** Alda Sabalic, Victoria Moiseeva, Andres Cisneros, Oleg Deryagin, Eusebio Perdiguero, Pura Muñoz-Cánoves, Jordi Garcia-Ojalvo

**Affiliations:** 1 Department of Medicine and Life Sciences, Universitat Pompeu Fabra, Barcelona, Spain; 2 Altos Labs, Inc., San Diego Institute of Science, San Diego, California, United States of America; Christian Albrechts Universitat zu Kiel, GERMANY

## Abstract

Identifying the genetic correlates of complex phenotypes is a challenging task. Methods coming from the field of complex networks can help finding such molecular patterns, by revealing statistical associations among groups of genes that correlate with the phenotype. Here we study cellular senescence, a complex cell state whose molecular underpinnings are still under active investigation. We analyze cell type–resolved RNA sequencing data obtained from injured muscle tissue in mice, with a network-based approach that merges eigenvector centrality feature selection and community detection. Our analysis identifies genetic markers that had not been associated with senescence so far, which are validated with existing single-cell RNA sequencing data in a different type of tissue. The identified key genes belong to transcriptional pathways associated with established hallmarks of senescence, and thus can be interpreted as molecular correlates of such hallmarks. The method proposed here could be applied to any complex cellular phenotype even when only bulk RNA sequencing is available, provided the data is resolved by cell type.

## Author summary

Senescence is a key manifestation of aging at the cellular level, caused by damage incurred by cells in time. In spite of their wide-ranging implications on how our multicellular bodies age, senescent cells are very challenging to identify due to their complex nature: many different aspects of cells are affected by this cellular state. This complicates defining clear criteria that help us decide whether a cell is senescent or not. In this paper, we propose a computational pipeline that enables us to identify a small subset of genes associated with senescence. The method combines two approaches commonly used in the study of networks, community detection and node centrality, and applies them to gene expression data obtained from the muscle tissue of mice after damage. The results obtained can contribute to establish the molecular correlates of a complex cellular state such as senescence.

## 1 Introduction

Cellular damage is usually correctly repaired in younger organisms, but it increasingly leaves traces in older individuals. Thus, as cells age, they tend to accumulate damage. One of the direct consequences of this process is cellular senescence, a phenomenon resulting from unresolved molecular damage. Senescence is a stable and permanent state of growth arrest in which cells are unable to proliferate [1]. It can occur in healthy cells that experience a chronic damage response, involving either direct damaging of the DNA or events like telomere or oncogenic mutations [2]. The affected cells undergo an irreversible cell cycle arrest but remain metabolically active rather than dying, which limits the damage’s effects [3]. Senescent cells are characterized by the transient expression of cell cycle inhibitors (e.g., p16^INK4a^, p21^CIP1/WAF^), DNA damage with double-strand breaks, chromatin alterations, erosion of the nuclear envelope, metabolic alterations such as oxidative stress, and a hypersecretory nature, the so-called senescent-associated secretory phenotype (SASP), composed by an heterogeneous range of growth factors, pro-inflammatory proteins, and matrix proteinases, which together alter the cellular state [4,5].

Senescence can occur in healthy cells that experience a damage response, involving either direct DNA damage, events like telomere shortening or oncogene activation, or other types of molecular damage and dysfunction, such as ER stress or mitochondrial dysfunction [6,7]. However, cellular senescence is not exclusively a response to molecular damage. A distinct modality, programmed or developmental senescence, occurs as a normal, transient part of an organism’s development [8–10]. This shows that senescence is a very heterogeneous phenomenon that can be a beneficial physiological process, and not just a pathological outcome of damage.

Multiple lines of evidence show that senescent cells are directly implicated in causing age-related phenotypes. In studies in which senescent cells are genetically or pharmacologically removed, both rapidly and naturally aged mice stay healthy much longer, and, in some cases, even display signs of aging reversal [11,12]. The opposite process has also been proven to be true: introducing only a small number of senescent cells into young mice results in physical dysfunction [13]. Moreover, it was found that senescent cells persist for extended periods, which leads to their accumulation during aging [14].

Even though senescent cells have shown high expression of cell cycle regulators such as proteins p16 and p21, a universally accepted criterion for their identification is still lacking [15]. Techniques such as RNA sequencing (RNA-seq), both bulk and single-cell, can offer valuable insights by comparing the global transcriptional activity of senescent and non-senescent cells [16–18]. However, traditional data analysis approaches have not revealed clear transcriptional signatures so far, probably due to the fact that senescence is a highly complex phenotype with multiple cellular implications. Within that context, the aim of this study was to complement the standard bioinformatics approaches by using a network-based method, which enables us to perform a “last-mile” filtering of the set of analyzed genes, and to identify a small number of genes that are highly central in distinguishing the senescent from the non-senescent transcriptional profiles. To that end, we use senescent cells arising in muscle tissue of aging mice during regeneration, following chemically-triggered injury.

Adult mammalian skeletal muscle is a stable tissue with low turnover, yet it retains a powerful capacity for rapid and extensive regeneration after severe injury [19]. This repair process occurs in two distinct phases. First, the degenerative phase begins with the necrosis (death) of muscle fibers, typically caused by sarcolemma disruption. This damage leads to an influx of calcium, activating proteases that degrade the fiber, and triggers a critical inflammatory response. Neutrophils are the first immune cells to arrive, followed by macrophages, which clear cellular debris and help activate the subsequent repair. This is followed by the regenerative phase, which is driven by the activation and proliferation of muscle stem cells. These cells multiply to provide new myonuclei, then differentiate and fuse to repair damaged fibers or create new ones. We and others described that during skeletal muscle regeneration, senescent cells accumulate after injury and with age. In particular, we generated the first senescence atlas of regenerating muscle by developing a novel enrichment protocol and characterized the cell types that become senescent, revealing that fibro-adipogenic progenitors (FAPs) and myeloid cells are dominant senescent cell types creating an “aged-like” niche that impairs regeneration in both young and old mice [20].

Standard bioinformatics analysis are commonly used to identify global transcriptional patterns that potentially differ among cell types. These analysis usually consider large numbers of genes (even after filtering), which renders their results substantially prone to variability. Here we use two complex network approaches to identify a small set of prioritized genes that best distinguish senescence from non-senescence. Complex networks serve as a representation of interactions and connections between their elements (which in the context of this investigation are both genes and cell states [21–23]) and are increasingly being used to identifying disease biomarkers [24,25] and molecular mechanisms of cellular function [26]. Importantly, complex networks are usually characterized by a community structure [27–31], meaning that elements belonging to a community are highly interconnected to each other and therefore grouped together. Conversely, different communities are loosely associated with each other, and their elements are considered less similar. The community structure that arises from a network constructed using transcriptional data [32,33] can provide useful information about the transcriptional determinants of a complex cell state such as senescence.

## 2 Results

### 2.1 Dataset description

**Fig 1:**
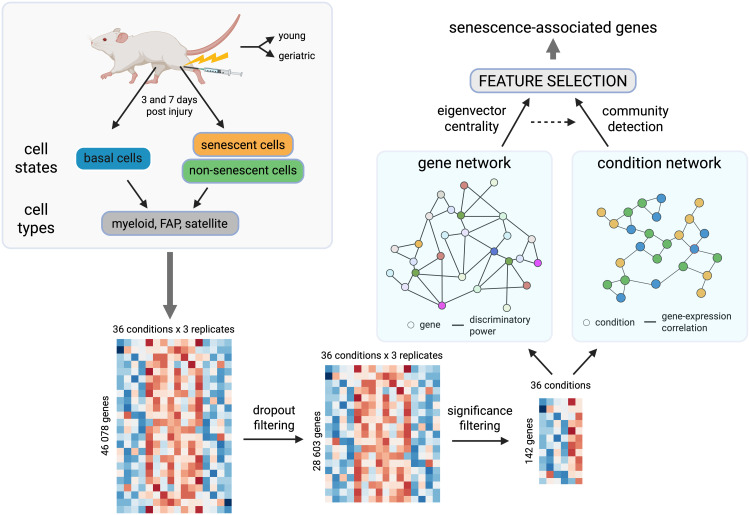
Workflow of the computational design. Muscle tissue was extracted from the injured and non-injured hindlimb of young and geriatric mice at 3 and 7 days post-injury. Basal cells were extracted from the non-injured limb, and senescent and non-senescent cells were taken from the injured limb. Three cell types of interest were isolated by FACS using well-established markers for myeloid cells, FAPs, and satellite cells. Senescent cells were further sorted using SPiDER-β-gal, as previously described [20]. Low input RNA-seq was performed for all three cell states: basal, senescent, and non-senescent, leading to initial gene expression matrix consisting of 108 samples each, with 46078 genes. This matrix was serially filtered to remove dropouts and non-discriminant genes. The final 142×36 matrix was then used to build two networks, one of genes and the other of conditions. Feature selection was then performed by combining eigenvector centrality of the gene network with community selection of the condition network. The process culminates in the identification of a small number of senescence-associated genes. Created in BioRender. Garcia-Ojalvo, J. (2026) https://BioRender.com/n1qw8gc.

Our study focuses on revealing the transcriptional regulation of cellular senescence during muscle repair and aging, by hypothesizing that cellular senescence can be discriminated via its gene expression profile. To that end, we analyzed the transcriptional profile obtained by bulk RNA-seq of distinct cell types. We used experimental data obtained by Moiseeva et al [20] from muscle tissue of healthy and injured mice. Cells from those tissues were separated into three cellular states: senescent, non-senescent and basal (Fig 1).

Data was obtained by performing experiments on 18 mature adult mice belonging to two age groups – 9 young (aged 3 months) and 9 geriatric (aged 284 months). The mice were subsequently divided into three pools, after which the left hindlimb was injected with cardiotoxin, causing massive damage to the skeletal muscle tissue [34]. Although the regenerative response happened almost immediately in both young and geriatric mice, senescent cells emerge after the necrotic phase of muscle regeneration, when the inflammatory response is present [20,35]. The damaged muscle tissue was extracted 3 and 7 days after the injury, representing early and late regeneration phases. The tissue was then processed, and the cell types were isolated by FACS (see Sec. 4.1). We focused on three different cell types because of their crucial role in muscle tissue regeneration - satellite cells (muscle stem cells), fibro-adipogenic progenitor (FAP) cells, and myeloid cells [36,37]. Each of the selected cell types was further categorized into three cell states: senescent or non-senescent, extracted from the injured (left) hindlimb, or basal, extracted from the non-injured (right) limb. RNA from the cells was then used for a low input RNA-seq analysis to determine the expression levels of a total of 46078 genes, including protein-coding genes, non-coding genes, and pseudogenes.

We thus considered three cell types and three cell states in both young and geriatric mice, at two different time points (3 and 7 days after injury). As each condition was replicated three times, this resulted in a total of 108 possible combinations, which represented the 108 conditions considered in the analysis. In this context, each of the 108 conditions is characterized by a 46078-dimensional vector, whereby each dimension corresponds to the expression level of a specific gene.

### 2.2 Preliminary filtering

**Fig 2:**
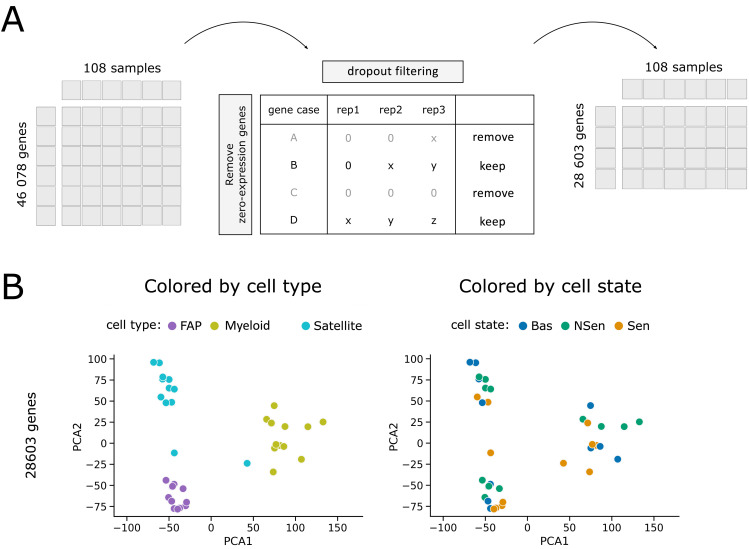
Dropout filtering. **(A)** The initial expression matrix consisted of 108 samples each defined by 46 078 genes. The 108 samples arise from 36 different cell conditions, each with 3 replicates. The first filtering consisted of averaging the expression of each gene among the 3 replicates of each condition and removing the ones whose expression was equal to 0 in at least 2 out of 3 replicates (gene cases A and **C)**. This combination of averaging and filtering led to an expression matrix of 36 samples (representing the different cell conditions) and 28 603 genes. **(B)** Principal component analysis of the remaining data, with cell conditions sorted either by cell type (left) or cell state (right).

RNA-seq is prone to exhibiting substantial technical noise, leading to dropout events in which a transcript is missing in a given replicate [38]. To avoid this issue, we averaged the expression of the non-zero replicates for each condition and kept only the genes whose expression was non-zero in at least 2 out of 3 replicates for at least one of the conditions. By doing so, we reduced the number of conditions to 36, and the number of genes to 28 603 (Fig 2A).

We next asked to what extent the resulting filtering gene set was able to identify the senescent phenotype. Given the large size of the data-set, we performed a dimensionality reduction via principal component analysis (PCA). Fig 2B shows scatter-plots of the different cell conditions for the first two principal components. The conditions are color-coded depending on either the cell type (left panel) or the cell state (right panel). As shown in the plots, the 28 603 genes resulting from the filtering procedure described above could discriminate well between the three cell types analyzed (left panel), but not at all between cell *states* (right panel). In other words, the selected genes are not sufficiently filtered to separate the senescent from the non-senescent phenotype.

**Fig 3:**
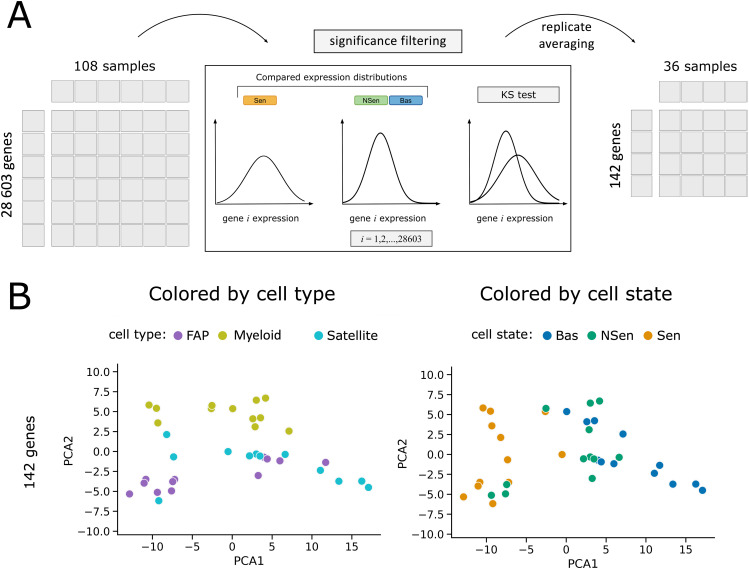
Significance filtering. **(A)** We compared the gene-expression distributions of the conditions between the senescent cell state and the non-senescent and basal states grouped together, for each one of the 28 603 genes. The distributions were compared by using the Kolmogorov-Smirnov test and applying a Bonferroni correction to the p-value of 0.05. A total of 142 genes passed the test. **(B)** Principal component analysis of the remaining data, with cell conditions sorted either by cell type (left) or cell state (right).

As our primary objective was to find the genes that can effectively discriminate between the three different cell states, rather than the cell types, further filtering was necessary. To that end, we conducted a Kolmogorov-Smirnov statistical test to compare the expression distributions of the senescent samples to the non-senescent and basal ones (Fig 3A). Since we use all three replicates for each condition, the sample sizes used in the Kolmogorov-Smirnov test are *n* = 36 for the senescence samples and *m* = 72 samples for the non-senescence ones. After applying the Bonferroni statistical test adjustment to the p-value of 0.05 and eliminating the genes whose distributions were not significantly different among the cell states, we reduced the initial number of genes to 142. To validate this filtering approach, we compared the genes identified by the KS test to those identified by a standard differential expression (DE) analysis between the senescent and non-senescent conditions, using the DESeq2 framework and a design matrix that accounts for all the experimental conditions (phenotype, cell type, age group, and time after injury). The results show that ∼98% of the genes resulting from KS filtering are also considered significant by the DE analysis (S2 Fig). We also compared all other experimental condition pairs (young vs geriatric mice, 3 vs 7 days post injury, and all three cell type pairs) using the DE analysis, and examined how the KS-filtered genes fared in those comparisons. We found that the KS-filtered genes were only statistically significant, according to the DE analysis, when comparing the senescent and non-senescent phenotype (S3 Fig). For other condition pairs, the KS genes mimicked the DE significance distribution, with most of them lying below the significance threshold. This indicates, in our opinion, that any potential confounding effect by age, or time after injury or cell type, is unlikely to influence the results of the KS filtering described above.

Next we averaged the expression levels of the different replicates of each condition (considering only the non-zero expression values in case B of Fig 2A), to work with a single expression level per condition. This led to a gene expression matrix of 36 conditions and 142 cells (Fig 3A, right). Interestingly, the strong reduction in the gene number eliminated the ability of the gene set to distinguish among cell types (Fig 3B, left panel). However, the data was now slightly more useful in distinguishing between cell states (compare the right panels of Figs 2B and 3B).

### 2.3 Ranking and filtering genes via the eigenvector centrality feature selection

**Fig 4:**
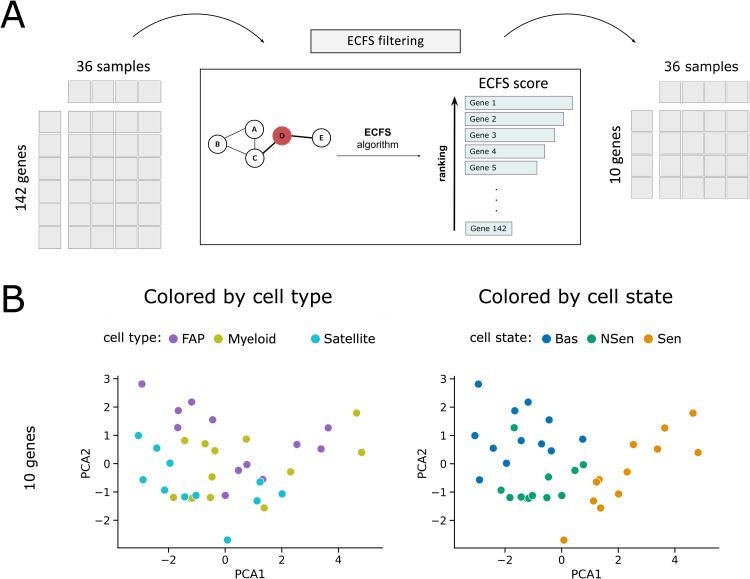
Ranking and filtering genes via eigenvector centrality feature selection. **(A)** The 142 genes that remained after filtering were ranked based on their importance in class separation using the ECFS algorithm. The top 10 ranked genes were retained, using the criteria discussed in Sec. 2.4. **(B)** Principal component analysis of the remaining data, with cell conditions sorted either by cell type (left) or cell state (right).

The preliminary filtering results showed that filtering the gene set enables a better separation between cell states (specifically, the senescent versus non-senescent phenotypes) at the expense of the discrimination between cell types (which is not a priority in our case). However, the cell-state separation still needed to be improved. We thus decided to focus further on the genes that are more strongly associated with the senescent phenotype. To that end, we implemented the eigenvector centrality feature selection (ECFS) algorithm [39] (Fig 4A). The ECFS algorithm is a network-based method that aims to determine the most relevant features in a given classification task (in our case, we chose to distinguish between the non-senescent cells and the rest). The algorithm uses a fully connected weighted network, where the nodes are represented by the features (in our case, the genes), and the edges represent how discriminative the associated features are for the classification. Subsequently, it evaluates the discriminatory power of all the features by calculating their eigenvector centrality scores. A detailed explanation of the use and implementation of the algorithm is given in Sec. 4.2.

After applying the ECSF approach, we obtained a ranking of the 142 genes in order of importance for the chosen class separation. The higher the eigenvector centrality score, the more influential the gene is in differentiating between the various conditions. We would like to use this ranking to perform a final selection of the genes that we expect to have a key role in separating the senescent from the non-senescent phenotype. However, the explicit values of the eigenvector centrality scores proved not to be useful in establishing a threshold that could define a set of selected genes, since they decreased too gradually. A different approach is thus needed for that purpose. To address this issue, we turned to a second network approach, namely community detection, and applied it to a network of cellular conditions, instead of the original network of genes (Fig 1). As explained in the next section, we built multiple versions of this network, with increasing numbers of genes according to their decreasing eigenvector centrality ranking, and chose the number of genes that led to a better separation between senescent and non-senescent phenotypes. This resulted in a set of 10 genes, for which the PCA results showed a clear improvement in the ability to distinguish the senescent phenotype from the other phenotypes. In what follows, we explain the way in which this number of genes was selected.

### 2.4 Network community analysis

**Fig 5:**
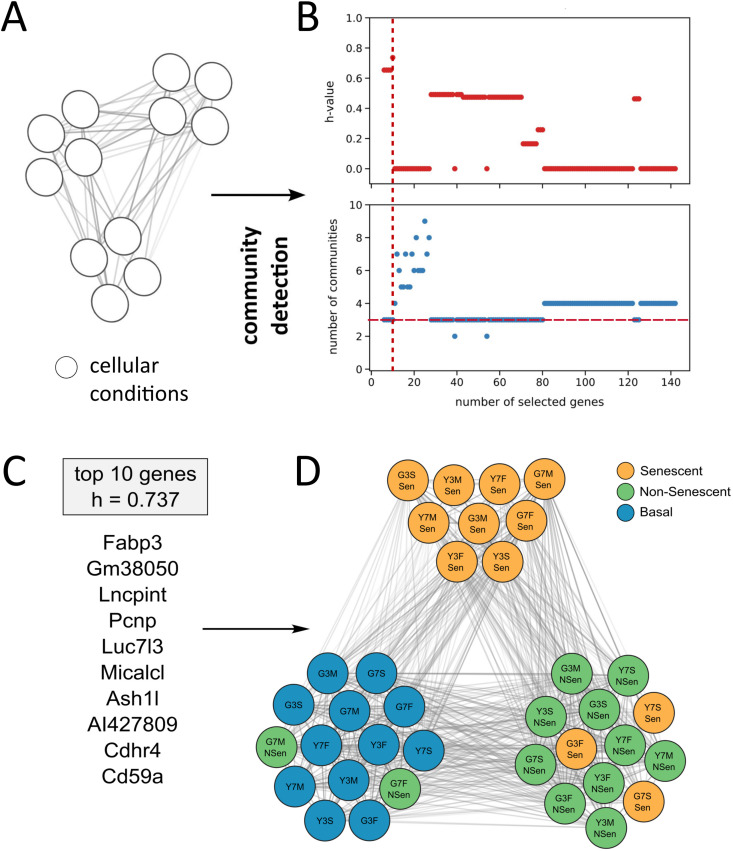
Network-community assessment. **(A)** Once the ECFS ranking was obtained we constructed cell-state networks, as described in the main text. **(B)** ECFS ranking performance for increasing number of genes, when comparing the non-senescent phenotype with the rest. The *h* score and the number of communities are plotted in the top and bottom panels, respectively. The best *h*-score of 0.737 was obtained when considering the first 10 genes. The vertical and horizontal red dashed lines correspond to the optimal *h* score and the three communities, respectively. **(C,D)** For that case we obtain the best community separation, represented here. The genes listed on panel C give rise to 3 communities, each one predominantly consisting of a single cell state –basal, senescent or non-senescent– (shown in blue, orange, and green, respectively). The node labels have the format A*n*B, where A is either Y or G, corresponding to the sample coming from a young or geriatric mouse, respectively, *n* is either 3 or 7, corresponding to day 3 or day 7 after the injury, and B is either M, F or S, corresponding to the myeloid, FAP or satellite cell type, respectively.

After ranking the genes with the ECFS algorithm, we proceed to build a network while considering an increasing number *N* of ranked genes, adding one gene at a time in the order of the ranking in each iteration of the method. The nodes of this network are represented by the 36 cell conditions, while the edge weights correspond to the Pearson’s correlation coefficients between the expression of the selected genes of the corresponding pair of conditions, rather than a binary value characterizing the presence or absence of an edge based on a certain cut-off (Fig 5A). Once the network was constructed, we identified its communities using the Louvain algorithm [40]. The process was repeated until all 142 genes were included. The number of communities obtained for increasing number of ranked genes included in the network is shown in the bottom panel of Fig 5B.

Ideally, the community detection algorithm should identify exactly three communities, with each cell state (senescent, non-senescent, and basal) belonging to its own individual community. Additionally, it was important to quantify the separation quality of the communities, i.e., how well the communities obtained with a given selection of genes discriminate between the cell states. For this, we introduced a goodness of separation measure based on Shannon’s entropy $H=-\sum_{i}p_{i}\ln(p_{i})$, where $p_{i}$ is the probability of occurrence of a specific event *i*, with the different events representing the grouping of a cell condition (senescent, non-senescent, and basal) to a specific network community. The entropy was computed with respect to both communities and cell states, and the total entropy $H_{tot}$ of a given community separation was obtained by summing the individual entropies. For a thorough description of this process along with an illustrative example, see Sec. 4.3.

Once we have computed the total entropy of the community separation, we now quantify the goodness of separation by defining the following quantity:


$$\begin{array}{c} h=\left\{ \begin{array}{c} \frac{H_{max}-H_{tot}}{H_{max}},\text{if }n=3\\ 0,\text{ if }n\neq 3, \end{array}\right. \end{array}$$ (1)

where *n* is the number of communities found by the community detection algorithm, and $H_{max}$ is the maximum possible value of the entropy, which corresponds to the case in which there is no clustering and each cell condition is in its own community (36 communities in total). Furthermore, the total entropy $H_{tot}$ is normalized by $H_{max}$, and its sign is inverted so that an *h*-value of 0 indicates maximum disorder, while a value of 1 indicates perfect separability of the three cell states, each belonging to its own community (see Sec. 4.3). Finally, we consider for assessment only the cases for which exactly three communities were detected. The values of the *h* score for increasing number of genes is shown in the top panel of Fig 5B.

The best cell state separation was obtained when the top 10 ranked genes from the ECFS algorithm output were considered (Fig 5B; the corresponding genes are listed in Fig 5C, and the corresponding network is shown in Fig 5D). Notably, one community contained 9 out of 12 senescent cell types but no other cell state (top orange cluster in Fig 5D). The other two communities are mostly basal, in one case (bottom left cluster, mostly blue, in Fig 5D), and mostly non-senescent, in the other (bottom right cluster, mostly green, in the same figure). The separation in those two cases is not perfect, however, with two non-senescent conditions appearing in the basal community, and three senescent conditions showing up in the non-senescent community. This may be explained by the fact that non-senescent cells display shared characteristics both with basal cells (as they have not yet developed any traits specific to senescence by the time the tissue was collected) and with senescent cells (as both of these states are extracted from the injured leg).

To test the robustness of the obtained separation, we performed a permutation test that consisted of randomly changing the labels of the 36 cell conditions 1000 times. Those artificial datasets then underwent the ECFS algorithm, and the community detection was applied on a network constructed with the 10 top-ranking genes. We found that it is significantly unlikely that such separation could be obtained by chance (p-value = 0.0019). This analysis is discussed in Sec. 4.4, and the corresponding distribution of randomly computed normalized entropy scores *h* is given in Fig11 below.

A final filtering of the gene list was performed by removing each one of the 10 genes obtained above one by one, and determining which of these removals resulted in a noticeable disruption of the community structure, as regards the separation of cell states. The results of this analysis are shown in Table 1. Furthermore, if only the six highlighted genes are considered for network construction and community detection, they produce the same separation among cell states as the 10 best-ranked genes.

**Table 1:** Effect of eliminating one by one each gene on the goodness of separation. Those genes whose absence noticeably disrupts the cell-state separation are highlighted.

Eliminated gene	*h* score
Fabp3	0.605
Gm38050	0.737
Lncpint	0
Pcnp	0
Luc7l3	0
Micalcl	0.737
Ash1l	0.737
Al427809	0
Cdhr4	0.737
CD59a	0.654

### 2.5 Comparison with other feature selection methods

**Fig 6:**
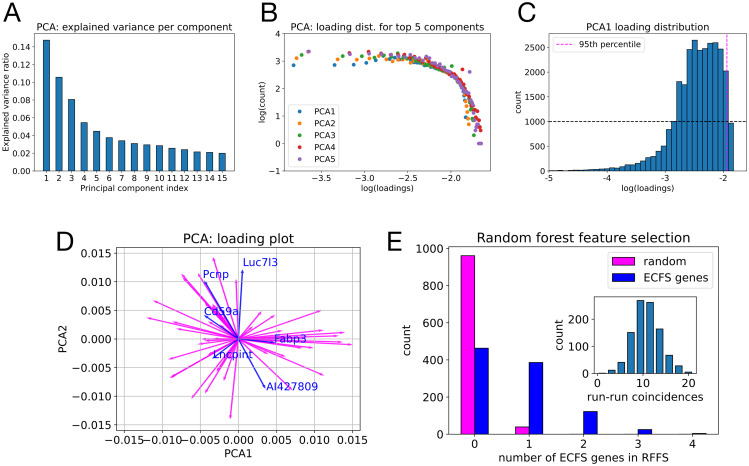
Contrasting with other feature selection approaches. **(A)** Fraction of variance explained by the different principal components for the transcriptomics dataset resulting from dropout filtering, see Fig 2. The first 15 principal components are shown. **(B)** Distribution of loading coefficients for the first 5 principal components. **(C)** Histogram of loading coefficients showing the contribution of all genes in the filtered dataset (28 603 genes) for the first principal component. The vertical dashed line corresponds to the 95% of the maximum loading coefficient. **(D)** Loading plot showing the contribution of the six genes found by the ECFS-based method (Table 1), in blue, and of 50 random genes, in magenta, to the first two principal components. **(E)** Histogram showing the number of realizations (out of 1000) in which the six genes found by the ECFS-based method (Table 1) appear in the top 200 genes identified by the Python scikit-learn random forest classifier, in blue, in comparison with six random genes used as control, in magenta. The inset shows the number of coinciding genes between pairs of realizations.

We have shown so far that combining network community analysis with eigenvector centrality ranking can constitute a useful approach for feature selection. It is natural to ask how this method compares with existing feature selection techniques. Answering this question will also allow us to biologically validate the results obtained. An immediate alternative approach to feature selection is principal component analysis, which we have used so far throughout the paper to evaluate the different filtering approaches used: simple dropout filtering (Fig 2B), significance filtering (Fig 3B), and finally our eigenvector centrality-based method (Fig 4B). We focus on the PCA analysis of the dropout-filtered dataset (28 603 genes). Fig 6A represents the variance of the dataset explained by the first 15 principal components. The figure shows a relatively slow decay of the explained variance for decreasingly ranked principal components.

We next asked how many genes contribute to the top principal components. Fig 6B shows that there are no subsets of dominant genes, since the distribution of loading coefficients drops quite rapidly for large loadings. The absence of fat tails in these distributions, similar for the top 5 principal components (represented by the different colored circles in the plot), indicates that there are no small subset of genes that dominate in feature space. This is confirmed when analyzing in detail the distribution, as shown in Fig 6C for the first principal component. This plot indicates that around 1000 genes have loading coefficients in the top 5% of all the genes in the dataset. We also quantified the contributions of the genes identified by our ECFS-based method to the first two principal components (Fig 6D, blue arrows), and found no qualitative difference with the contribution of genes selected at random (magenta arrows). Together, these results indicate that PCA is not an adequate way of selecting a small number of features (genes) to be associated with senescence. A similar conclusion can be reached by applying the method to the much smaller number of genes (142) resulting from the significance filtering of Fig 3B (see S1A-S1C Fig).

A more dedicated approach to feature selection is provided by random forest (RF) classification [41,42]. When applied to our dropout-filtered dataset, the method ranked the genes according to their ability to distinguish between classes (senescence and non-senescence –including basal–, in our case). We repeated the estimation process 1000 times and selected the top 200 ranked genes in each run. We observed low consistence between realizations, with only around 5% of the top 200 genes coinciding from run to run (Fig 6E, inset). Notably, at least one instance of the six genes identified with our ECFS-based method appears in more than half of the top 200 genes ranked by the RF feature selection algorithm (blue bars in Fig 6E), much more frequently than randomly selected genes (magenta bars). This can be interpreted as validation of the ECFS method: the six genes identified by the method repeatedly appear consistently among the much larger (and much less consistent) set of genes singled out by RF classification. When applying the random forest feature selection approach to the significance-filtered gene set (142 genes, Fig 3B), the results (Suppl. Fig S1D) show still a low consistence of the method from run to run, while the genes identified by ECFS keep appearing consistently among the top 50 RF genes (although in this case randomly selected genes also appear consistently, due to the small size of the pool –142 genes in this case–).

### 2.6 Validation with single-cell RNA sequencing data

**Fig 7:**
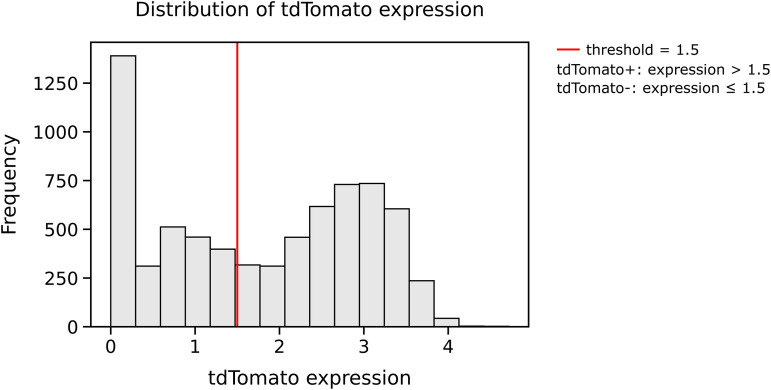
Distribution of p16 expression in single cells. The expression among all the considered cell types, as measured in linear scale by the fluorescent marker tdTomato, presents a bimodal distribution. The threshold for establishing p16+ and p16- cells is chosen to be 1.5. The code for this analysis and for the one shown in Fig 8 below can be found in the paper repository, https://github.com/dsb-lab/CellularSenescence.

To additionally test the relevance of our findings, we conducted a validation process based on single-cell RNA-seq data of normal and fibrotic liver and kidney tissue obtained by Omori et al [43]. That study used p16 as a marker of senescence, as senescent cells have an increased expression of that protein, at least during some part of their evolution [44]. The authors generated a transgenic mouse model in which cells that express p16 are permanently traced with the fluorescent marker tdTomato, thereby providing a lineage tracing reporter model for p16. We reanalyzed their data using genome assembly GRCm38 from Ensembl release 81 [45]. Following Omori et al [43], we split the population into two subgroups based on the expression of tdTomato, since according to that work high levels of tdTomato are indicative of high p16 expression. The distribution of tdTomato levels (in linear scale) is shown in Fig 7 for the liver data published in that work. Since that distribution was seen to be bimodal, we chose to split the cells simply into negative and positive cells (labeled p16- and p16+ below), by using a threshold value located near the minimum between the two expression peaks (vertical red line in the figure). The results shown below are not sensitive to this choice, as long as the two peaks are kept separate.

**Fig 8:**
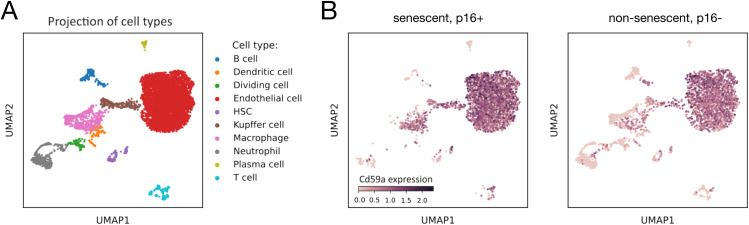
Expression of CD59a in p16+ and p16- cells. **(A)** UMAP projection of all the cell types considered. **(B)** The cells are classified into two groups based on their tdTomato expression. Cells with tdTomato expression larger than 1.5 are considered p16 + , whereas cells that have the expression of tdTomato ≤1.5 are labeled as p16-. A predominant presence of increased CD59a expression is observed in the p16 + cells for macrophages and Kupffer cells, indicating that CD59a could be a marker of senescence in those cell types.

The analysis above allowed us to establish a ground-truth separation between senescent cells (p16 + , with tdTomato expression above the threshold) and non-senescent cells (p16-, with tdTomato expression below the threshold). The cells were then projected in a low dimensional UMAP representation of their transcriptome for the two cell states, as shown in Fig 8A. We then superimposed the expression of one of the six genes identified with our analysis, CD59a (which has only recently been associated with senescence, see Section 3 below), on this UMAP representation. As can be seen when comparing the two panels of Fig 8B, CD59a exhibited a clear enrichment in the senescent cell populations, larger than other traditional markers. Strikingly, the best categorization can be observed among Kupffer cells and macrophages, which are the myeloid cells of the liver and the only cell types common with our dataset. Other cell types, such endothelial and plasma cells, showed a poorer match between p16+ and CD59a. The fact that the cell types used for validation come from a liver dataset shows that our results can be generalized to other tissues. Our approach, however, performs better for cell lineages similar to those abundant in regenerating skeletal muscle, such as myeloid cells and probably mesenchymal cells.

## 3 Discussion

Some of the six genes highlighted in Table 1 were found to be directly associated with senescence. One of the genes most relevant for the separation was Lncpint, a p53-induced long intergenic non-coding transcript. Removing this gene from the analysis resulted in a complete disruption of the otherwise well-defined clusters, losing senescent cell separation completely. This indicates the crucial role of this transcript in the differentiation of the senescent cell state. There have been numerous reports about the role of Lncpint in various biological processes ranging from DNA damage responses [46], cell cycle and growth arrest [47], cellular senescence [48], cell migration [49] and apoptosis [47]. These findings are consistent with our results.

Three other genes fully disrupt the separation between senescent and non-senescent genes when they are not taken into account: Pcnp, Luc7l3, and Al427809. Of these, Pcnp and Al427809 have not been associated with cellular senescence so far, to our knowledge, although the human ortholog PCNP has been recently associated with phenotypic behaviors that overlap with senescence, such as cell proliferation, viability, and apoptosis [50]. Similarly, Luc7l3 has been recently shown to have a protective role against genome instability in human cells, leading to senescence when absent [51], suggesting a similar role in mouse cells. Luc7l3 is also commonly linked to the spliceosome, which is relevant in our case, since growing evidence implicates altered splicing to cellular senescence and aging [52].

Another highlighted gene, Fabp3 (fatty acid-binding protein 3), is involved in the intracellular transport of long-chain fatty acids. Fabp3 has been reported to be upregulated in senescent cells, together with numerous other lipid-transport and lipid metabolism genes [20]. In general, lipid uptake plays a role among the generally accepted hallmarks of senescence, which include cell cycle arrest, resistance of apoptosis, metabolic and morphological changes, and highly secretory phenotype (SASP) [53]. Lipids are essential for each of these features [54]. Furthermore, an accumulation of lipid droplets in senescent cells has been reported in various studies [55–57].

Finally, our single-cell validation, discussed in the previous section, has highlighted one of the genes identified in this study, CD59a, as a major factor distinguishing senescent from non-senescent cells. In consonance with this result, this gene has recently been associated with senescence. CD59a protects host cells from complement-mediated lysis by blocking the formation of the membrane attack complex (MAC). In the context of senescence, CD59a may contribute to the survival and persistence of senescent cells against immune clearance, especially considering that senescent cells upregulate SASP factors related to the complement. Therapy-induced senescent cells have been shown to upregulate CD59a (as well as factor H, another complement inhibitor) while simultaneously upregulating factors related to the activation of the complement [58]. In contrast, mice knocked down for CD59a showed complement dysregulation and increased senescence markers [59].

**Fig 9:**
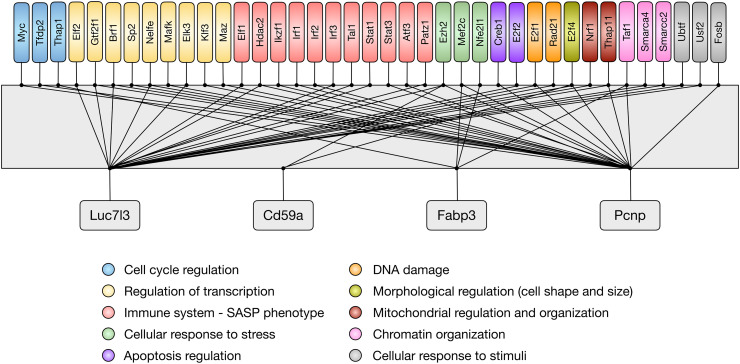
Transcription factors regulating the identified genes and their role in senescence. The transcription factors from the most relevant protein-coding genes identified by our approach are grouped by color, representing the pathways they are involved in, as established by their Gene Ontology (GO) annotations. We use MyGene.info to query the GO database (https://mygene.info). Information about transcriptional interactions is obtained from SCENIC [62]. The python script to query the GO database and identify the transcription factor categories can be found in the paper’s public software repository, https://github.com/dsb-lab/CellularSenescence.

To provide an additional assessment of the other genes involved in the cell state separation, we conducted an upstream analysis of their transcription factors (TFs). Specifically, out of the six essential genes found to separate the clusters, 4 were identified as protein-coding. The TFs regulating those genes were extracted from the Gene Regulatory Network database (GRNdb) [60]. Given that the analyzed samples were extracted from muscle tissue, we used the TF-target regulations for mouse muscle available in the database. Subsequently, we assessed the implication of a particular TF in a pathway using the KEGG Pathways database [61]. We then grouped the TFs according to their involvement in the pathways associated with the established hallmarks of senescence. Many of the identified TFs were involved in pathways linked to several hallmarks of senescence such as cell cycle, secretory phenotype, cellular response to stress, apoptosis resistance, DNA damage, morphological alterations, accumulation of mitochondria, chromatin organization, and cellular response to stimuli (Fig 9).

In summary, the goal of this work was to increase our understanding of the transcriptional regulatory landscape of cellular senescence in tissue regeneration. To that end, we conducted an analysis that leans on network-theory concepts such as community detection and eigenvector centrality. Network centrality measures have recently been introduced as tools to identify genes associated with diseases such as cancers [63,64] and mental disorders [65], or to identify the relationship between genome architecture and transcriptional programs [66]. In these approaches, eigenvector centrality is used to assess gene influence in the underlying molecular networks. In contrast, here we use eigenvalue centrality specifically for feature selection. This is accomplished by combining centrality ranking of gene discrimination networks with community analysis of cell state networks. Our approach enables us to identify a small group of genes that most clearly discriminate between phenotypes (in our case, senescence versus non-senescence).

Specifically, our analysis resulted in a set of six genes that are able to almost completely separate the senescent phenotype from the non-senescent and basal ones. While we observed some mixing within the identified clusters, we attribute this to the transcriptional similarity between cell types in injury conditions independent of their cellular state, and to the known heterogeneity of the senescent cell populations between time points and ages, which can hide any differences with their non-senescent counterparts. On the other hand, it is worth noting that in our community analysis there are no senescent cells present in the basal cell community, and vice versa. This result supports the hypothesis that basal and senescent cells have a sufficiently different transcriptional signature.

Our results underscore that, from a transcriptional point of view, senescence is a heterogeneous process characterized by a variety of molecular hallmarks. In search of these hallmarks, we identified a gene set that underlies the senescent phenotype. The relevance of the identified genes was assessed in terms of their transcriptional roles, which were found to correspond to several established phenotypical hallmarks of senescence.

A limitation of our work is that our analysis of senescent cells is mainly based on a specific context (e.g., acute muscle regeneration). Thus, the markers found may not reflect all types of senescent cells seen in other organs or stress conditions. We mitigated this by validating the data in another *in vivo* senescent cell dataset comprising the liver, and observing consistent behavior for key genes like CD59a. However, further research is necessary to empirically confirm whether these genes actively drive or sustain senescence.

## 4 Materials and methods

### 4.1 Experimental data

All experimental data were obtained by Moiseeva et al [20]. Briefly, single cells were isolated by flow cytometry sorting according to their cell type (senescent, non-senescent and basal) and processed for RNA sequencing. Senescent cells were identified using specifications that include criteria consistent with subsequently published standardized guidelines [67,68]. The full experimental protocol, together with the RNA extraction procedure, is described in the above-mentioned paper.

### 4.2 Eigenvector centrality feature selection

The eigenvector centrality feature selection (ECFS) algorithm [39] is based on mapping the given feature selection problem to a weighted graph describing the relation between features of interest and a classification task. In our analysis, the features are the expression of the genes that are expected to distinguish (classify) among different cell states (senescent, non-senescent, and basal). For details on the mathematical approach underlying this method, see the original publication by Roffo and Melzi [39]. We use their original implementation of the algorithm as a computational Matlab package, which we wrap in Python to interface with the rest of our computational pipeline. For details of the entire pipeline, including the connection with the original Matlab implementation of the ECFS algorithm that we use, see our paper repository at https://github.com/dsb-lab/CellularSenescence.

### 4.3 Measuring the goodness of separation

**Fig 10:**
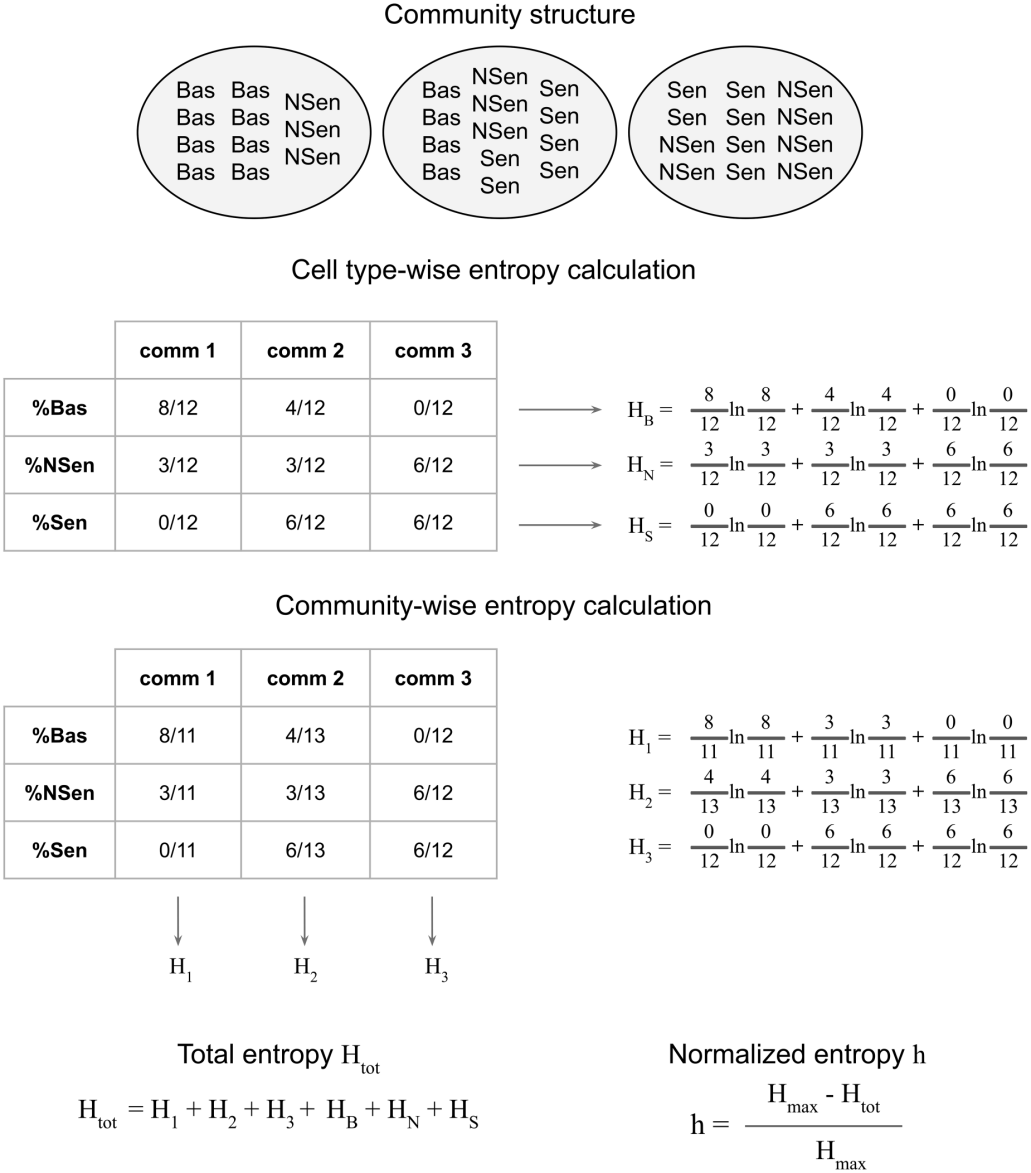
Entropy calculation. To obtain the total entropy $H_{tot}$, first, we have to calculate the individual entropies, both cell type-wise and community-wise. Since a total of 12 senescent, 12 non-senescent and 12 basal cells were present in our analysis, in the cell type-wise entropy computation, the probability $p_{i}$ is defined as the probability of a cell found in a community to belong to a given cell state. In the community-wise entropy calculation, the probability $p_{i}$ is defined by the ratio of cells belonging to a specific cell state to the overall number of cells within that community. The total entropy $H_{tot}$ is calculated by summing all the individual cell type-wise and community-wise entropies. The normalized entropy value *h* is obtained by normalizing the total entropy $H_{tot}$ with the maximum possible entropy $H_{max}$ that corresponds to the case when each cell belongs to its own separate community.

As the data of our analysis consists of three different cell states (basal, senescent and non-senescent), the goodness of separation score is calculated only in the cases when the number of communities *n* detected by the community detection algorithm is exactly 3. Fig 10 provides an illustrative example of how the goodness of separation is determined. First, the probability of occurrence of a given cell state $p_{i}$ was defined for every cell state. Taking into account that the 36 analyzed conditions include 12 senescent, 12 non-senescent, and 12 basal cells, in the cell type-wise entropy calculation, each entry $p_{i}$ corresponds to the probability of a cell present in a community to belong to a given cell state. Therefore, when summing the values of $p_{i}$ for each row, a total probability of 1 is obtained (first table in Fig 10).

In the community-wise calculation, each entry $p_{i}$ represents the ratio of cells belonging to a cell state and the total number of cells in that community. In this case, the total probability of 1 is achieved by summing up the entries $p_{i}$ for each column, as shown in the lower table in Fig 10. Subsequently, Shannon’s entropy defined as $H=-\sum_{i}p_{i}\ln(p_{i})$ is calculated for each of the cell states and cell types. The total entropy $H_{tot}$ is defined as the sum of the individual entropies. To keep the entropy values in the interval between 0 and 1, a normalized entropy value *h* is computed. The normalization was done by taking into account the maximum possible entropy $H_{max}$ that corresponds to the case in which each cell belongs to its own community. The normalized entropy value is given as:


$$\begin{array}{c} h=\left\{ \begin{array}{c} \frac{H_{max}-H_{tot}}{H_{max}},\text{if }n=3\\ 0,\text{ if }n\neq 3, \end{array}\right. \end{array}$$ (2)

where *n* is the number of communities found by the community detection algorithm and $H_{max}=-3\ln\frac{1}{12}$.

### 4.4 Statistical validation

**Fig 11:**
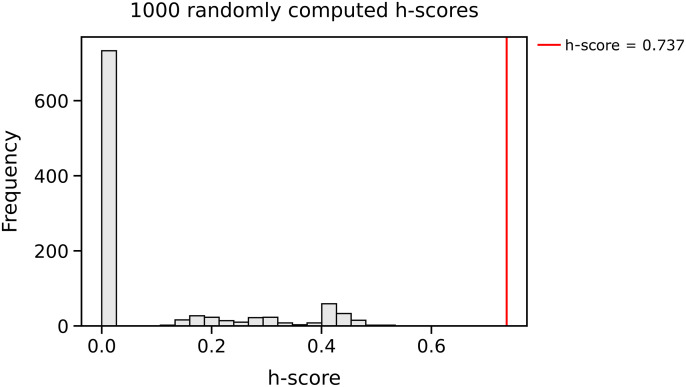
Histogram of randomly computed *h*-scores. The *h*-scores represented in the histogram were computed by permuting the cell conditions labels. The artificially created labels were sorted into 2 classes, one consisting of non-senescent cells and the other of senescent and basal cells. The ECFS algorithm was performed for the classes, and the corresponding networks with the communities were computed. The *h*-score obtained for the 10 best-performing genes was extracted. The described procedure was repeated 1000 times and the corresponding *h*-scores are shown in the histogram. The red line represents the original maximum *h*-score obtained by our separation (*h* = 0.737). The p-value for our obtained *h*-score to be part of this distribution was found to be 0.00019.

To assess the robustness of the separation obtained by the 10 selected genes, a randomized permutation test was performed. The test consisted in generating 1000 datasets by permuting the labels of the 36 cell conditions. Each dataset was first put through the ECFS algorithm to obtain the ranking of the genes, after which the network was formed and community detection was performed for the top 10 ranked genes. By doing so, we obtained a distribution of the calculated normalized entropy scores *h*. The normalized entropy score from the original dataset *h* was then compared to the distribution by a Kolmogorov-Smirnov test. The test results showed that it is significantly unlikely to obtain such separation by chance, giving a p-value of 0.0019. The distribution of *h*-scores together with the *h*-score from the original dataset are shown in Fig 11.

## Supporting information

S1 Fig Analysis of the significance-filtered data with other feature-selection approaches. (A) Fraction of variance explained by the different principal components for the transcriptomics dataset resulting from significance filtering, see Fig 3. The first 15 principal components are shown. (B) Histogram of loading coefficients showing the contribution of all genes in the filtered dataset (142 genes) for the first principal component. (C) Loading plot showing the contribution of the six genes found by the ECFS-based method (Table 1), in blue, and of 50 random genes, in magenta, to the first two principal components. (D) Histogram showing the number of realizations (out of 1000) in which the six genes found by the ECFS-based method (Table 1) appear in the top 50 genes identified by the Python scikit-learn random forest classifier, in blue, in comparison with six random genes used as control, in magenta. The inset shows the number of coinciding genes between pairs of realizations.
(PDF)

S2 Fig Comparison between KS and DE filtering. Scatter plot showing the statistical significance (y axis) versus the fold change (x axis) for all genes remaining after the dropout filtering (28 603 genes), resulting from a differential expression analysis of the senescent versus non-senescent phenotypes (grey symbols). The genes resulting from KS filtering are highlighted in red. The horizontal dashed line represents the significance threshold *p* = 0.05. The analysis was performed using the software package PyDESeq2, a Python implementation of the DESeq2 algorithm [69].
(PDF)

S3 Fig DE statistical significance distributions for all condition pairs. Distributions of adjusted p-values (in logarithmic scale), as obtained from differential expression analyses of all pairwise condition comparisons (as described in the panel titles). The blue bars represent the distributions of all the dropout-filtered genes (28 603 genes), while the red bars show the distributions of the KS-filtered genes. All distributions were normalized to unit area for easy comparison. The Wasserstein distances between the red and blue distributions in each case are shown in the grey inset titles.
(PDF)
