# Supplementary figures and images for: Cell-type resolved transcriptional network analysis of in vivo cellular senescence following injury
Source: PLoS Comput Biol. 2026 Jun 22;22(6):e1014429. doi: 10.1371/journal.pcbi.1014429 (PMC13309044; doi:10.1371/journal.pcbi.1014429)

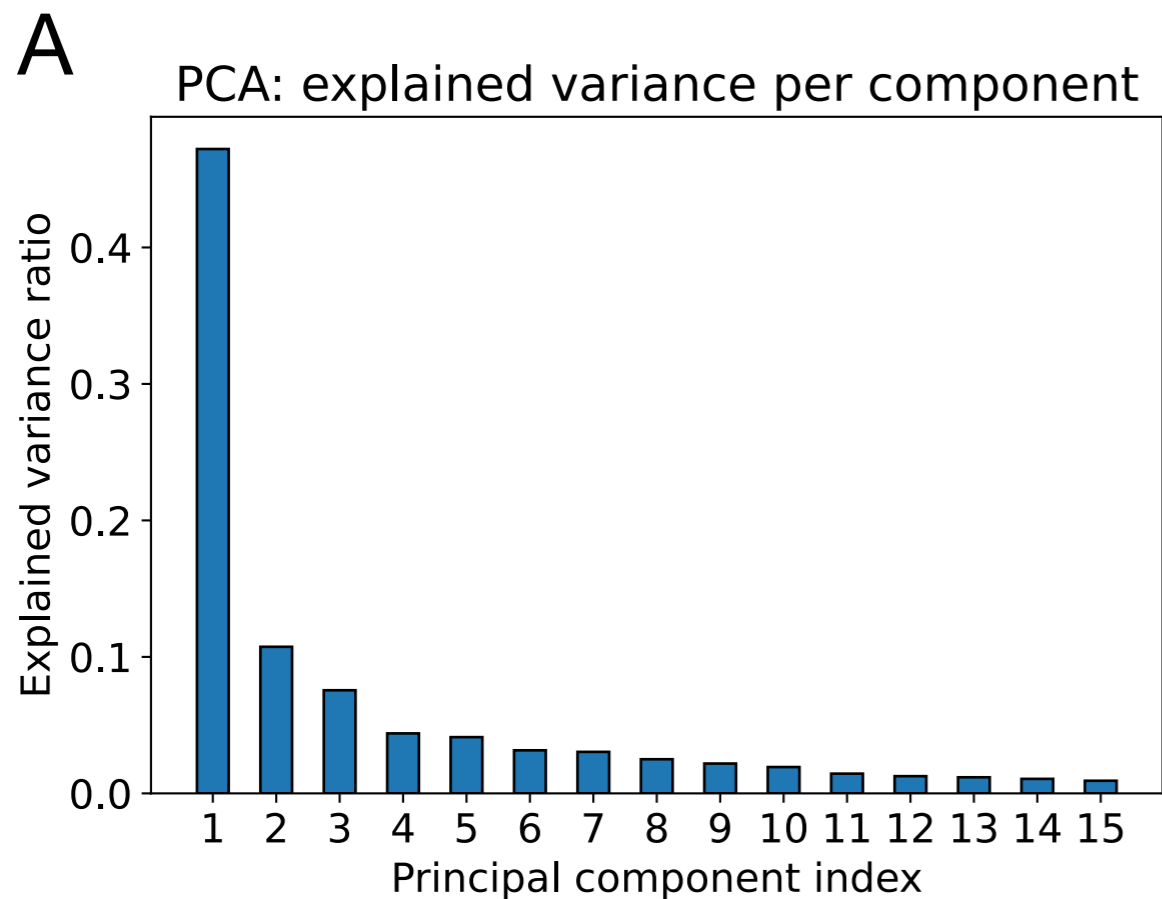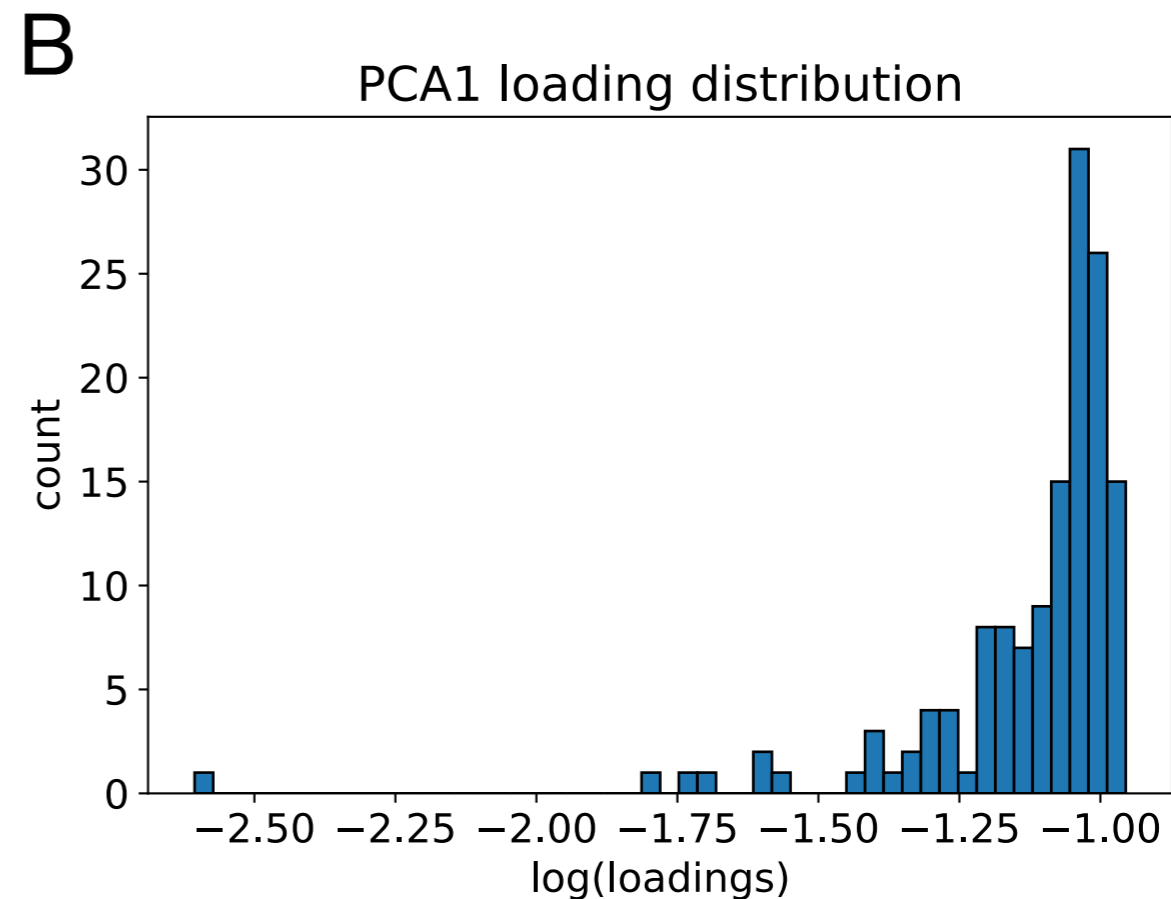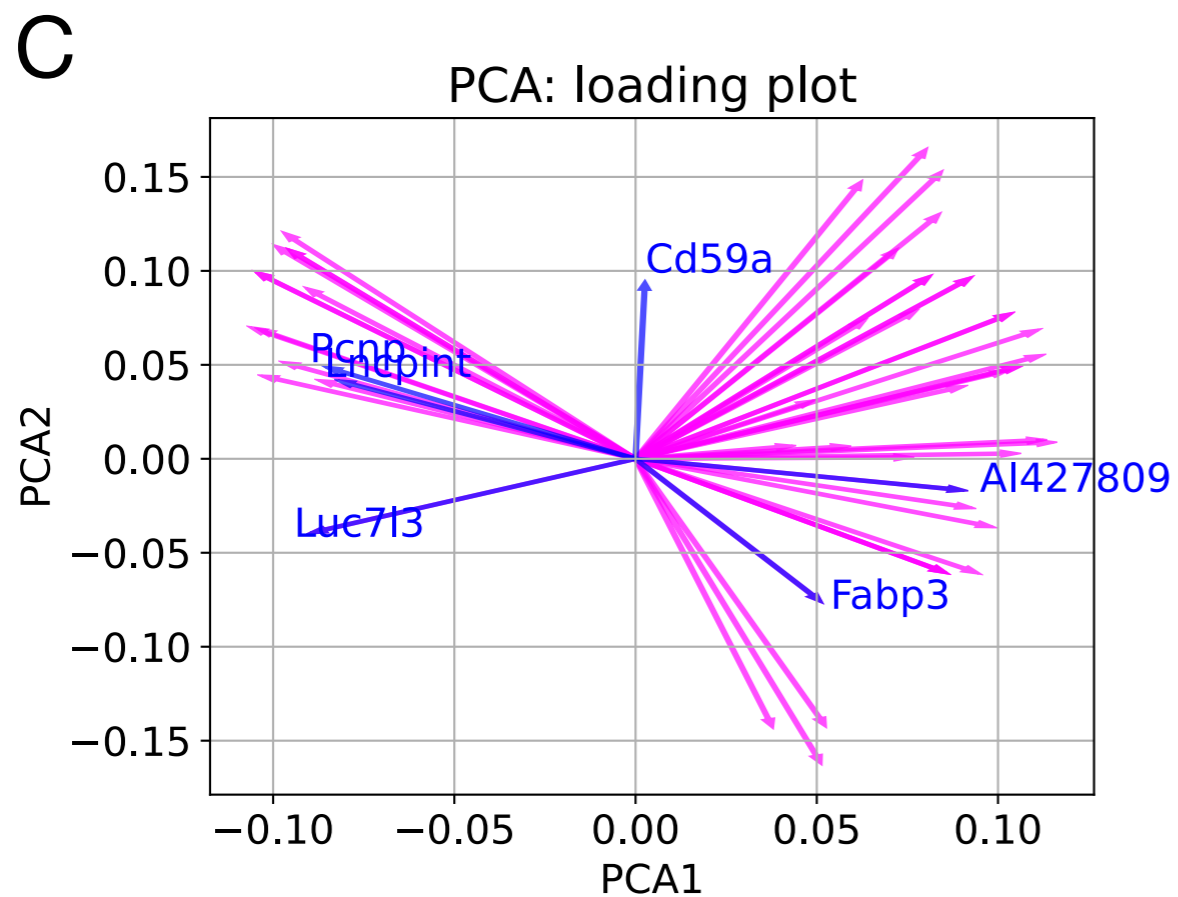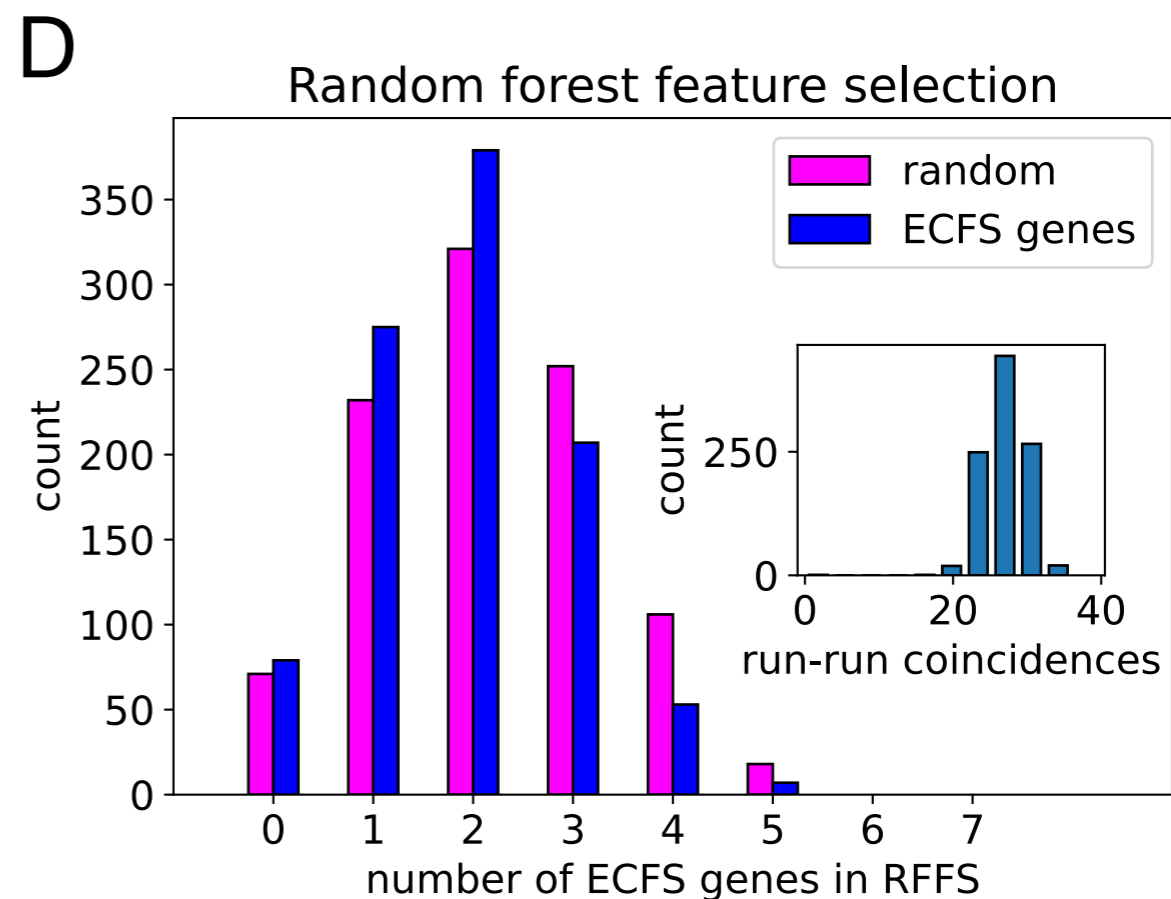

Supplement: S1 Fig — (A) Fraction of variance explained by the different principal components for the transcriptomics dataset resulting from significance filtering, see Fig 3. The first 15 principal components are shown. (B) Histogram of loading coefficients showing the contribution of all genes in the filtered dataset (142 genes) for the first principal component. (C) Loading plot showing the contribution of the six genes found by the ECFS-based method (Table 1), in blue, and of 50 random genes, in magenta, to the first two principal components. (D) Histogram showing the number of realizations (out of 1000) in which the six genes found by the ECFS-based method (Table 1) appear in the top 50 genes identified by the Python scikit-learn random forest classifier, in blue, in comparison with six random genes used as control, in magenta. The inset shows the number of coinciding genes between pairs of realizations. (PDF) [file pcbi.1014429.s001.pdf]

# senescent vs non-senescent

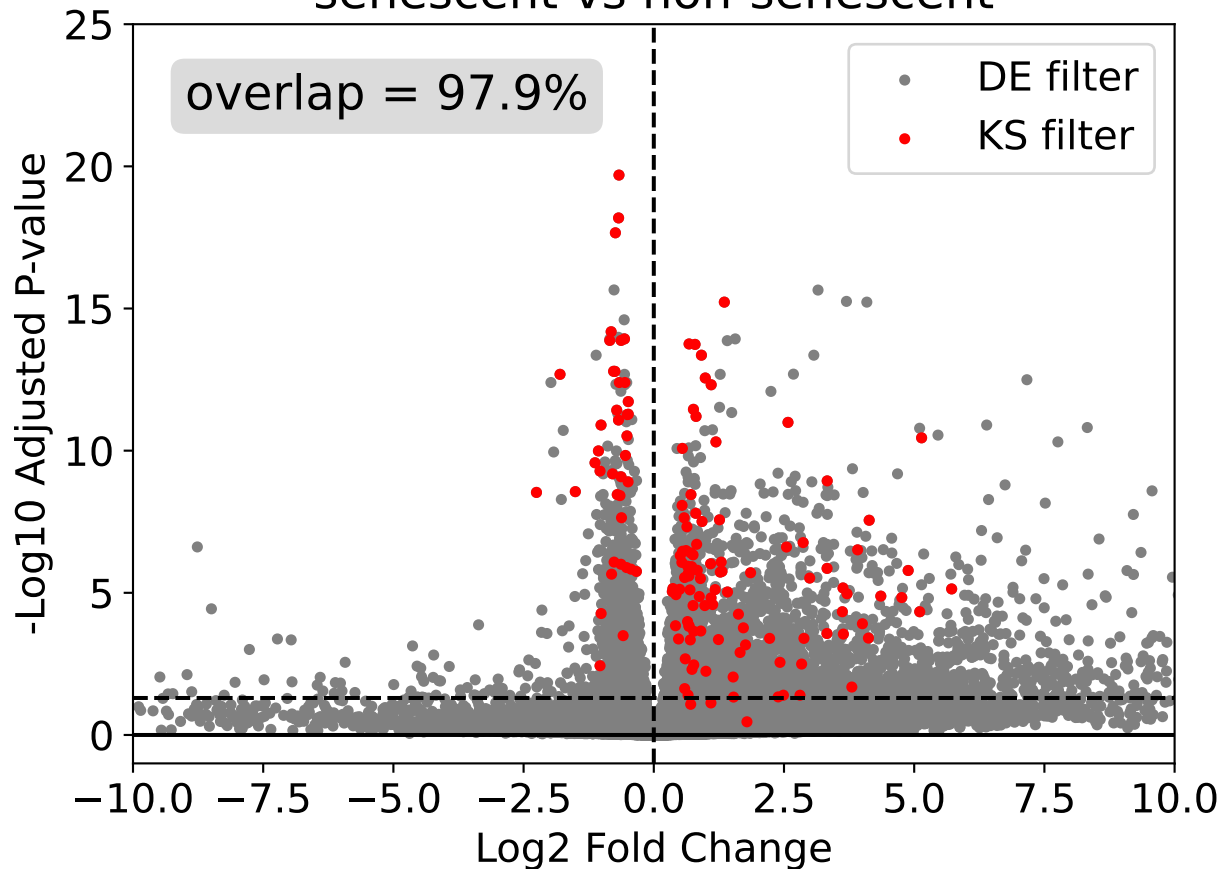

Supplement: S2 Fig — Scatter plot showing the statistical significance (y axis) versus the fold change (x axis) for all genes remaining after the dropout filtering (28 603 genes), resulting from a differential expression analysis of the senescent versus non-senescent phenotypes (grey symbols). The genes resulting from KS filtering are highlighted in red. The horizontal dashed line represents the significance threshold p = 0.05. The analysis was performed using the software package PyDESeq2, a Python implementation of the DESeq2 algorithm [69]. (PDF) [file pcbi.1014429.s002.pdf]
