# Supplementary material for: Cell-type resolved transcriptional network analysis of in vivo cellular senescence following injury
Source: PLoS Comput Biol. 2026 Jun 22;22(6):e1014429. doi: 10.1371/journal.pcbi.1014429 (PMC13309044; doi:10.1371/journal.pcbi.1014429)
Supplement: S3 Fig — Distributions of adjusted p-values (in logarithmic scale), as obtained from differential expression analyses of all pairwise condition comparisons (as described in the panel titles). The blue bars represent the distributions of all the dropout-filtered genes (28 603 genes), while the red bars show the distributions of the KS-filtered genes. All distributions were normalized to unit area for easy comparison. The Wasserstein distances between the red and blue distributions in each case are shown in the grey inset titles. (PDF) [file pcbi.1014429.s003.pdf]

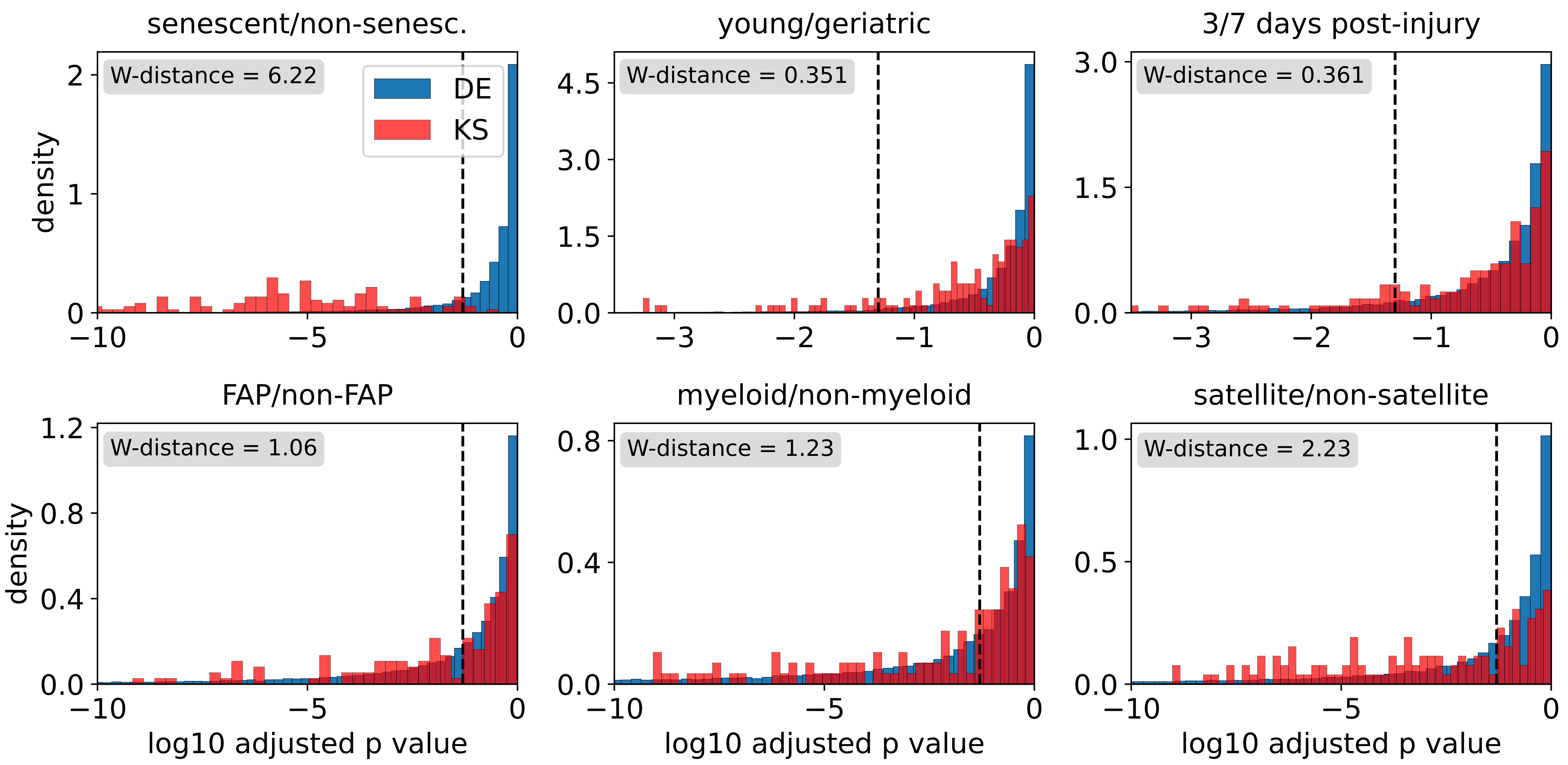
senescent/non-senesc.
W-distance = 6.22
DE
KS
density
log10 adjusted p value
young/geriatric
W-distance = 0.351
3/7 days post-injury
W-distance = 0.361
FAP/non-FAP
W-distance = 1.06
myeloid/non-myeloid
W-distance = 1.23
satellite/non-satellite
W-distance = 2.23
