# Supplementary material for: Cell-type resolved transcriptional network analysis of in vivo cellular senescence following injury
Source: PLoS Comput Biol. 2026 Jun 22;22(6):e1014429. doi: 10.1371/journal.pcbi.1014429 (PMC13309044; doi:10.1371/journal.pcbi.1014429)
Supplement: Attachment — Submitted filename: senescence_response_to_reviewers.pdf [file pcbi.1014429.s005.pdf]

We deeply appreciate the thoughtful and constructive comments of the reviewers. In what follows we respond to the different points raised, with the reviewer comments highlighted in *blue italics*, and our responses in regular typeface. We also describe the changes made to the manuscript to address those points, using the line numbers of the resubmitted manuscript (a version with tracked changes is included in this resubmission).

Reviewer #1

The study addresses a biologically significant and computationally challenging problem by identifying robust molecular markers for molecular characterization of cellular senescence. The methodology is clearly described, and the code is made available via GitHub. The validation by using independent scRNA-seq data enhances confidence in the biological relevance and generalizability of the results.

The manuscript is well structured and written with a concise message.

We thank the reviewer for their positive opinion.

Major Comments

1. While the results are compelling and rather convincing, the ms would benefit from a more detailed comparison between their ECFS and community detection approach with other methodologies like WGCNA, random forest feature selection or detailed PCA and ICA analyses. This would help the readers to contextualize the novelty and utility of their pipeline. This said, a more comprehensive description of the PCA analysis would be also interesting in terms of the explained variability of the different PCs, how the other PCs may discriminate the different cell states and if there is biological meaningful information in the PC loads/weights.

We thank the reviewer for this comment. We certainly agree that a more comprehensive description of the PCA approach was necessary, since this analysis is an important through line in the paper. We now include a detailed discussion of the PCA results, focusing on the analysis of the dataset after dropout filtering (Fig. 2). The analysis is aimed at comparing the feature selection capabilities of our method with that of the PCA approach, as suggested by the reviewer. The outcome of this analysis is shown in a new Results subsection (new section 2.5, “Comparison with other feature selection methods”). Briefly, the results show that PCA is not able to associate a small subset of genes to the senescent phenotype, since a large fraction of the 28000+ genes considered have a similar contribution to the top principal components. The results are shown in the new Figure 6. The analysis is also applied to the significance-filtered dataset (Fig. 3), leading to a similar conclusion. Those results are now shown in a new supplementary figure (Fig. S1).

We also implemented, following the reviewer’s suggestion, a random forest (RF) feature selection approach to our dataset. The results, also shown in the new Figs. 6 and S1 and discussed in the new section 2.5, indicate that the gene ranking obtained by this method is not consistent between different random runs of the algorithm. Interestingly, the genes obtained from our ECFS method do appear consistently within the top ranked genes produced by the RF method, much more frequently than random genes. We consider this an additional validation of

our results. As for the WGCNA method suggested by the reviewer, we did try to implement it, but the results were hard to interpret in terms of feature selection, since this method is aimed more specifically at identifying functional connectivity patterns of gene expression. Obtaining sets of genes from these patterns would require, in our opinion, additional assumptions that would complicate the interpretation of the results. For this reason we decided to focus our effort on the PCA approach (which we were using already) and the random forest classification (which is more easily comparable to our ECFS-based ranking).

2. The biological interpretation of how the gene candidates relate to senescence could be more extensive. Particularly, Cd59a would benefit from deeper discussion of potential mechanisms or prior evidence in senescence biology.

We agree with the reviewer that a more detailed discussion on CD59a was missing in the previous version of the manuscript. We have now included this in lines 347-357 of the Discussion section, which includes two new references. Of the remaining genes resulting from our analysis, Lcnpint and Fapb3 have been clearly linked with senescence in the past, as described in the first two paragraphs of the Discussion section. The remaining three genes, Pcnp, Luc7l3, and AI427809, have not been directly associated with senescence, although the first two play roles in human cells that functionally overlap with senescence. This is now described in the new second paragraph of the Discussion section, lines 330-338, where we now cite two new references.

Furthermore, some more detailed discussion on the limitations of the study would allow to put the results in a broader biological context. While the confirmation in liver data is interesting, the potential context could be discussed further.

We thank the reviewer for this suggestion. We have added a paragraph on the limitations of the study at the end of the Discussion section, lines 397-402.

3. Some more clarification on the experimental set-up is needed. Were some experiments performed for this study or was it all done in relation to ref 23? Accordingly to the statement "There are no primary data in the paper." no experiments were performed, but then the FACS sorting description in Sec. 8.1 is confusing. If experiments were done for this study, the ethics should be also specified.

We apologize for the confusion. No new experiments were done for this study. We have rewritten Sec. 8.1 (now Sec. 4.1), and changed its title and that of Sec. 2.1 (whose contents have also been somewhat rephrased) to clarify this. We have also redesigned Fig. 1 and changed its title and caption, to clarify the design of this particular study.

Minor comments

- Page 2: replace "-" with "," or add a space in "… between their elements –which in the context of this investigation".

- Page 7: use lower case "s" in "is explained in the next Section.".

We thank the reviewer for pointing out these typos, which are now corrected in the revised version of the manuscript.

- Page 9: Something is missing in the sentence "Similar to the mixing of basal and non-senescent cells, this might be because both of these cell states are extracted from the injured leg."?

This sentence was indeed very confusing. We have rephrased the entire paragraph (lines 224-232) to clarify our interpretation of the communities obtained.

- Page 12: Please clarify the sentence "It is worth noting that although the cell types used for validation originated from an entirely distinct tissue from those in our analysis, we could use our results to find the senescent cells that overlapped with our dataset, indicating that our conclusions for myeloid cells might be generalizable."

We have rewritten this sentence, please see lines 316-319 at the end of Sec. 2.6.

- It would be nice to have a README in the github repository giving the context of the repository and maybe also mention the corresponding python version. For me it worked at least with python 3.9.17.

We have fully reorganized our repository to make it clearer for the readers, and to add the new code for the additional PCA analyses and the random forest feature selection. We have also included a README giving the context of the software, and mentioning the version of Python in which it can be run.

- It would be interesting to see the distribution of the ECSF scores to have a better intuition on the gene selection.

The distribution of ECFS scores was unfortunately not informative to help decide on where the cutoff should be made, because these scores decrease gradually with no clear features that allow us to establish a threshold. That is the reason why we performed the community analysis of the network of cellular conditions. We very much appreciate the reviewer's comment, which made us realize that this point was not explained clearly in the previous version of the manuscript. We now describe this in detail at the end of Section 2.3 (lines 174-187), and also in the new Fig. 1.

- What is the reasoning for 1.5 as a threshold in splitting the dTomato population in Figure 7?

In contrast with the previous point, in this case the distribution of tdTomato levels (Fig. 7) does allow us to choose a threshold that best separates the cells into p16+ and p16- subpopulations. Specifically, we choose the threshold as the value located around the minimum between the two expression peaks. We tested that the results were not sensitive to this choice, as long as the separation between the peaks is maintained. We now clarify this point in Section 2.6 (lines 296-304).

Besides these comments, the manuscript could be a very valuable contribution to PLoS Computational Biology when the points above are addressed accordingly.

We thank the reviewer for their positive opinion and constructive comments.

<u>Reviewer #2</u>

The authors have used cell-type resolved bulk RNA-seq with eigenvector centrality analysis and community detection (Louvain) to compress senescence vs. non-senescence transcriptomes into a small discriminative set of six genes that separates cell states across FAPs, myeloid cells, and satellite cells during muscle regeneration after injury. Additional analysis is used to quantify the robustness of the separation analysis. As far as I know these approaches are sound and original and the findings.

We would like to thank the reviewer for their appreciation of the soundness and originality of our results.

While the analysis highlights some potentially novel candidate markers of senescence in the context of skeletal muscle regeneration and their transcriptional regulatory networks, I am less certain about the originality and significance of these findings given that several major studies in the field appear not to have been cited.

We are also very thankful to the reviewer for pointing out the need to cite and comment on several recent publications. In what follows we respond to the different suggestions separately.

Ogrodnik et al, "Guidelines for minimal information on cellular senescence experimentation in vivo". Cell 2024;187(16):4150-4175. This paper describes minimum information for cellular senescence experimentation in vivo that the authors should acknowledge and confirm that they followed.
Suryadevara et al, "SenNet recommendations for detecting senescent cells in different tissues". Nat Rev Mol Cell Biol. 2024;25(12):1001-1023. This article includes recommendations for detecting senescent cells in based on literature reported senescence markers in 14 tissues in mice and humans. It seems highly relevant but is not cited or discussed.

These articles describe recently proposed guidelines that we already used when generating the data analyzed in this manuscript (Moiseeva et al, "Senescence atlas reveals an aged-like inflamed niche that blunts muscle regeneration", Nature 2023, pp. 169–178; ref. 20 in the revised bibliography). Briefly, isolated cells were considered senescent when they exhibited several biological markers: induction of cell cycle inhibitors, decreased cell proliferation, erosion of the nuclear envelope, DNA damage, pro-secretory phenotype, metabolic changes, oxidative damage, and lysosomal alterations.

We do consider the papers mentioned by the reviewer relevant, but we did not cite them because our paper is previous to them and follows stricter criteria. Besides the indicated markers, senescent cells were depleted using two independent methods—genetically with the p16-3MR model and pharmacologically with Q+D. In both cases, reducing senescent cells led to faster muscle regeneration, as shown by histological and functional assessments. The results were shown to be reproducible, as different treatment schemes improved regeneration at

various ages. Also, the findings were extended to models of chronic injury and other types of injury (freeze-thaw) and micro-damage (needle injury).

Nevertheless, we thank the reviewer for pointing out these relevant, standard-setting papers, which we should definitely cite, and are doing so in the revised version of this manuscript (new refs. 67 and 68).

Li et al, "Multiomics and cellular senescence profiling of aging human skeletal muscle uncovers Maraviroc as a senotherapeutic approach for sarcopenia". Nat Commun. 2025;16(1):6207. This paper reports the first senescence atlas profiling senescence in human skeletal muscle.

This article uses an approach to study senescence *in silico* to try to enrich populations in scRNA-seq experiments. However, we consider that their approach cannot ensure a true identification of "bona fide senescent cells", as they do not follow the guidelines provided in the other articles mentioned by the reviewer or in our previous article (ref. 20 in the revised bibliography). On the other hand, we agree that it is worth citing this article in the context of the senescent-associated secretory phenotype (SASP), and have now done so in the introduction section (new ref. 5).

Walter et al, "Transcriptomic analysis of skeletal muscle regeneration across mouse lifespan identifies altered stem cell states". Nat Aging. 2024;4(12):1862-1881. Although focused more specifically on stem cell states, this paper identifies molecular markers of age-related renerative potential.

Although this article by the Cosgrove group is highly relevant and describes, by *in silico* characterization, senescent-like muscle stem cell subsets within injury zones in aged muscles, we did not find any similarity relevant to our results, since as the reviewer says, that work is centered on stem cell states. On the other hand, we agree that it is worth citing this article in the context of the relevance of transcriptional approaches to the study of senescence, and have now done so in the introduction section (new ref. 18).

<u>Reviewer #3</u>

Sabalic et al. report novel targets of cellular senescence in a skeletal muscle injury model. The paper primarily focuses on the development of computational methods to identify senescent cells within a mixed cell population, rather than presenting new biological findings. However, a general definition of senescent cells, including well-known biomarkers, should be provided.

We thank the reviewer for this request. As mentioned above, since the dataset used here stems from our Nature article (ref. 20 in the revised bibliography), where we extensively describe the subject, we kept our descriptions quite brief in the first version of the manuscript, for which we apologize. We have now expanded our definitions in the Introduction section, lines 36-51.

Additionally, the use of cells identified as senescent in aged humans or model organisms—rather than those resulting from artificially-induced muscle injury—may be necessary to justify the methodological approach.

Since this study is predominantly based on a chemically induced muscle injury model, both the overall scope and the title should more accurately reflect this focus instead of adopting a broadly generalized view.

Although our model uses cardiotoxin to induce injury, senescent cells appear as a secondary response to the regeneration process. For example, the myeloid populations originate from monocytes that infiltrate the tissue after injury. Thus, while the injury is triggered chemically, the appearance of senescent cells during regeneration is not. Therefore, the findings can be applied to various models of muscle injury. Supporting this, our previous study extended the results concerning the pharmacological inhibition of senescent cells to other models such as micro-puncture, freeze-crack, and the muscular dystrophy model (mdx) (ref. 20 in the revised bibliography).

In any case, to our knowledge there are no published transcriptomic datasets that isolate senescent cells from humans, and few have performed transcriptomics after isolation in mouse models. Therefore, our dataset is as comprehensive as possible. Nevertheless, we acknowledge the reviewer's remarks and have modified the title and abstract of the paper, and introduced several comments throughout the manuscript, to emphasize the focus of our work. In particular, we now explicitly state in the Introduction (lines 69-70) the model used, thereby refining the overall scope of the paper, as requested.

This consideration should also be integrated into the Introduction section, given that the model system was previously reported by the co-authors (Moiseeva et al., Nature 2023, doi: 10.1038/s41586-022-05535-x). Because the earlier paper employed a very similar mouse muscle injury model for both bulk and single-cell RNA sequencing, it is important to highlight the biological and methodological differences and the novel aspects of this current study.

Our previous article (ref. 20 in the revised paper), where the datasets used here were generated, describes senescent cells as key components of skeletal muscle that inhibit regeneration by creating an inflamed, fibrotic environment through the SASP. That study primarily used bulk transcriptomics to create the first comprehensive molecular and functional atlas of senescent cells in regenerating muscle. However, we only used scRNA-seq as a confirmatory tool to identify the more abundant senescent cell populations, and lacked a thorough, unbiased analysis that we have now conducted. The combination of an eigenvector centrality analysis of the gene network and a community analysis of the associated condition network presented here, proves to be more efficient in ranking genes associated with the senescent state, and enables a more targeted approach, in comparison with standard RNAseq analysis methods. This enhanced targeting aspect is reflected in the ability of the method to identify a small number of genes that strongly separates the transcriptomic landscape of senescent and non-senescent cells. We have modified the abstract and fully redesigned Fig. 1 to emphasize the specific characteristics of our approach that make it different from standard scRNAseq approaches.

Similar bioinformatic approaches using eigenvector centrality have been published for RNA-seq network community analysis by multiple groups, although none have specifically focused on senescence or muscle injury (Pashaei et al., NAR 2025, doi: 10.1093/NAR/GKAF609; Sarmah

and Bhattacharyya, J Biosci 2024, doi: 10.1007/s12038-024-00437-8; Wei et al., LSA 2023, doi: 10.26508/LSA.202302253; Liu et al., iScience 2018, doi: 10.1016/J.ISCI.2018.08.002). If the authors' methodology is primarily computational, they need to clearly articulate what makes their approach novel or more advantageous compared to these existing methods.

We thank the reviewer for pointing out this literature. Although these works do use eigenvector centrality in their pipelines (together with other centrality measures), they in general do so to assess the influence of genes within specific molecular networks, either protein-protein interaction networks (Pashaei et al, 2025), gene correlation networks (Sarmah and Bhattacharyya, 2024; Wei et al, 2023) or genome architecture networks (Liu et al, 2018). In contrast, our method uses eigenvalue centrality specifically for *feature selection* at the phenotypic level. This is accomplished by combining centrality ranking of *gene discrimination networks* with community analysis of *cell state networks*. We believe that this unique approach is better suited to identify the genes that most clearly discriminate between phenotypes (in our case, senescence versus non-senescence). We appreciate the reviewer's request of clearly articulating the novelty of our approach with the above-mentioned existing methods, and do so now in the Discussion section (lines 374-382). We have also modified the abstract and fully redesigned Fig. 1, as mentioned above, to emphasize the specific features of our approach.

Given that the utility of the current approach for identifying novel and generalizable senescence markers is considered, the analysis should be extended to more published datasets, coupled with biological validation.

As mentioned above, very few transcriptomic datasets (besides our atlas in ref. 20) isolate senescent cells *in vivo* in mouse models. We have used one of these datasets, derived from a different tissue, injury model, and senescent cell isolation strategy, to validate our approach (Fig. 7), obtaining results consistent with our analysis. Validating the results experimentally, *in vivo*, is in our opinion currently out of the scope of this article. On the other hand, we agree with the reviewer that the text was implicitly making unwarranted claims about the biological generality of the results. We have thus revised the manuscript in order to clarify the biological focus of our work. In particular, we have modified the title of the paper and rephrased parts of the text, including the abstract, the introduction, and the discussion sections. We hope that these changes will help reframe the generalizability of our claims.

Currently, the manuscript lacks clarity throughout; a thorough revision is recommended to emphasize the novel methodological and biological contributions.

We thank the reviewer for this comment. To address this issue we have extensively revised our manuscript, reorganizing its structure and adding clarifications of the method (including a thoroughly revised Fig. 1 describing the workflow of our computational pipeline) and its biological implications throughout the text.

Other Issues:

1. The first paragraph of the Introduction should adopt a broader perspective, as cellular aging is inherently heterogeneous. For example, some senescence pathways are not triggered by

molecular damage, whereas DNA damage remains an important cause—either directly or indirectly—of senescence. This may be a writing issue, as heterogeneity is acknowledged later in the manuscript.

We have substantially revised the Introduction to address this comment of the reviewer, for which we are really grateful. In particular, we now discuss the heterogeneous character of senescence early on (lines 44-51 of the Introduction section).

2. In relation to the above, it would be more effective to include an overview of the muscle injury model within the context of senescence research, given that this is the primary focus of the study.

We are also thankful for this request. We have now described the process of skeletal muscle regeneration and the use of this process in the context of senescence research, in lines 71-85 of the introduction.

3. While citing many review articles can be helpful, the authors should incorporate more primary research articles where relevant to strengthen their arguments.

We agree with the reviewer on this point. In this resubmission we have addressed this comment by adding new references to primary literature to support a variety of issues and claims.

4. The Experimental Data section should specify the sources of key materials, such as cardiotoxin and the mouse strain used, to ensure reproducibility. This basic information is currently lacking.

We apologize for the misunderstanding. All experimental data originate from ref. 20. Although we described the FACS protocol for the reader's information, it was not clear that this was performed in our previous work. We have clarified this in the Materials and Methods section (section 4.1), where we clearly refer now to ref. 20 (previously ref. 23) as the source of our data. We have also fully redesigned Fig. 1 to focus the attention of the reader in the computational pipeline, instead of the experimental implementation.

5. The statement, "We focused on three different cell types because of their crucial role in muscle tissue regeneration—satellite cells (muscle stem cells), fibroadipogenic progenitor (FAP) cells, and myeloid cells"—raises questions about their specific roles and the rationale behind selecting these cell types. Clarification is needed, particularly since the focus on these cells may be specific to this experimental model rather than applicable to cellular senescence broadly.

We thank the reviewer for this comment. We have now updated several parts of the manuscript to highlight the origin of our datasets and how, in our senescence atlas of regenerating muscle (ref. 20 in the revised bibliography), we characterized the cell types that become senescent. In particular, we now mention that fibro-adipogenic progenitors (FAPs) and myeloid cells are the most abundant senescent cell types, creating an "aged-like" niche that impairs regeneration driven by muscle stem cells in both young and old mice (lines 82-85 in the Introduction). Although this dataset might reflect aging and disease, it lacks other cell types that become senescent in different tissues and conditions. Accordingly, to acknowledge the reviewer's

remarks, we have changed the manuscript title to emphasize our focus on tissue regeneration after damage.

6. The manuscript should specify which gene database was used for RNA-seq analysis (e.g., GENCODE, REFSEQ, or others). Although the authors mention a total of 46,078 genes, the specific database and version used should be clearly indicated, as gene counts can vary between releases.

We used the GRCm38 genome assembly from Ensembl release 81. We now mention this in Section 2.6 (lines 296-297).

7. Providing clearer information about the genes identified in Table 1 and Figure 3 would be helpful. Notably, the color-coding legend in Figure 3 appears to use the same blue for both cell cycle and apoptosis resistance, which could be confusing. Furthermore, since transcription factors are depicted hierarchically, a label like "gene expression" is too broad; the database used for functional annotations should be cited. For example, there appears to be no match for Elf1 in mouse or human KEGG pathways, despite the authors' statement. Additionally, the phrase "The pathways shown are related to established senescence" in the figure legend would benefit from specific citations or source references.

We very much appreciate this comment from the reviewer, since it highlights several deficiencies in the original version of Fig. 6 (which is the figure we believe the reviewer was referring to, and which is now Fig. 9 in the revised manuscript). In response to these comments, we have redesigned the figure with a clearer color coding, and changed the label "gene expression" to make it more specific (although we appreciate the new label is still general, it corresponds to the categories identified by our pipeline). We now mention in the caption of Fig. 9 the database used for functional annotation, and removed the phrase mentioned by the reviewer, since it was redundant with the main text, where the comment is properly put into context. We also include in the software package the script used to identify the annotations given in the figure; this is also mentioned in the revised figure caption.

8. In Figure 7, the graph appears to represent a histogram, but it is unclear what scale is being used. Since tdTomato expression is intended to report p16 levels, clarification is needed. The cited Cell Metabolism paper describes tissues in terms of p16^high^ or p16^low^, reflecting their differing distributions of p16. The authors should clearly explain what they did in the current study to reassess the previously reported p16 data (Figures 7 and 8).

We apologize for our lack of clarity in this part of the manuscript. We now clearly explain in the text (section 2.6, lines 297-304) our reassessment of the data obtained by Omori et al in the cited paper. We have also revised the software repository associated with this manuscript (https://github.com/dsb-lab/CellularSenescence), clarifying in particular the Python notebook in which this analysis was performed. We have also revised the caption of Fig. 7 to clarify this point and to refer to the repository.

9. Regarding the computational implementations, did the authors use the MATLAB code provided in the cited paper on the eigenvector centrality ranking algorithm (Roffo and Melzi,

2016)? The mathematical formulations in Sections 8.2–8.3 are already presented in that paper, unless the authors introduced modifications. The current manuscript provides no information on how the method was actually implemented—whether as a computational package or as executable resources—which is a significant omission.

We are also very thankful to the reviewer for this comment. The mathematical formulation of the eigenvector centrality feature selection approach presented in the previous version of the manuscript was indeed redundant with respect to the original publication. We have thus removed it from the revised manuscript, and replaced it with a reference to that work. We did use the Matlab code from the original publication, inserted into our computational pipeline via a Python interface, which is now described in Section 4.2, lines 416-421.
