# Supplementary material for: Cell-type resolved transcriptional network analysis of in vivo cellular senescence following injury
Source: PLoS Comput Biol. 2026 Jun 22;22(6):e1014429. doi: 10.1371/journal.pcbi.1014429 (PMC13309044; doi:10.1371/journal.pcbi.1014429)
Supplement: Attachment — Submitted filename: senescence_response_to_reviewers_2.pdf [file pcbi.1014429.s006.pdf]

We very much appreciate the comments of the reviewer. In what follows we respond to the two points raised, with the reviewer comments highlighted in *blue italics*, and our responses in regular typeface. We also describe the changes made to the manuscript to address those points, using the line numbers of the resubmitted manuscript (a version with tracked changes is included in this resubmission).

Reviewer #3

A minor point first but in Figure 3 Significant filtering -- it appears the same as the 'discriminating filtering' shown in Figure 1. Filtering (and RNA-seq in general) is using statistical criteria (in this case the KS test). For this, using one term will reduce confusion.

We thank the reviewer for noticing this inconsistency in our terminology. We have now removed the expression "discriminant filtering" from figure 1, and substituted it by "significance filtering", in accordance with the term used in the rest of the paper.

More importantly, however, I must question whether the Kolmogorov-Smirnov test is really appropriate to compare the means of gene 'i' expression between sample groups with small sample size. In a total of the 36 samples without counting replicates, the sample size of each group could be around 10-20 (looks like 12 from the right panel of Fig 3B), considered too small below the generalized effective size range of 50 and 1000 for KS test. Although the authors applied Bonferroni correction, it tries to control overall Type I errors but it does not mitigate this issue. Therefore, it is advisable to find an alternative that tolerates small sample sizes better than KS test. In the same token, I wonder if a negative binomial method may simply suffice without separating the two filtering steps during bulk RNA-seq analysis as the authors used in Ref. 20. Simply, the use of ranking by the degree of differential expression available in the DEG list generated previously by EdgeR, may work similarly without raising the sample size issue above. Have the authors already tried it? Since the authors' main point of this manuscript is about application of the eigenvector centrality feature selection algorithm developed by another group earlier for network community analysis, the goal of RNA-seq analysis may be relatively peripheral. However, it is important to find a confidence in selected approach. By choosing a different test appropriate for small sample sizes in the second filtering step (or whole in RNA-seq data analysis), I believe that the manuscript requires a revision. Depending on the outcome, results and discussion (as well as methods) may also need adjustments.

This comment made us realize that our description of the significance filtering step was not correct, for which we apologize. Specifically, we applied the Kolmogorov-Smirnov (KS) test to *all* the replicates of our study, not to the average expression of the different conditions. This increases the sample sizes to 36 and 72 for the senescent and non-senescent conditions, respectively, instead of the 12 to 24 perceptively inferred by the reviewer from Fig. 3B. In our opinion these sample sizes are sufficient for the validity of the KS test. For that reason we chose to use this test instead of alternative methods such as the one suggested by the reviewer. We believe that this approach does not lead to an over-estimate of significant differences in our case, since the method only identifies 142 genes as being expressed differently between the two groups, out of the initial 28,603 genes analyzed (less than 0.5% of the genes studied). Furthermore, the resulting genes are further validated biologically in terms of the relevant literature of senescence, as discussed in Section 3. In any case, the reviewer's comment highlights a clear shortcoming in the previous version of

the manuscript, related to how we were describing our use of the KS test. To clarify this issue, we have modified Figs. 1-3 to make clear that the outcome of the dropout filtering is a 28,603×108 matrix, not a 28,603×36 one, and that the reduction to 36 conditions only happens after the significance filtering is performed. We have also rewritten parts of Section 2.2 (lines 135, 149-151, and 154-157 in the manuscript with tracked changes) to clarify this issue. We strongly appreciate this comment by the reviewer.
