# Supplementary material for: Cell-type resolved transcriptional network analysis of in vivo cellular senescence following injury
Source: PLoS Comput Biol. 2026 Jun 22;22(6):e1014429. doi: 10.1371/journal.pcbi.1014429 (PMC13309044; doi:10.1371/journal.pcbi.1014429)
Supplement: Attachment — Submitted filename: senescence_response_to_reviewers_3.pdf [file pcbi.1014429.s007.pdf]

PCOMPBIOL-D-25-01276R2

“Cell-type resolved transcriptional network analysis of in vivo cellular senescence following injury”

PLOS Computational Biology

RESPONSE TO REVIEWERS #3 AND #4

We are extremely thankful to reviewer #3 for their continuous guidance and for not giving up on our manuscript, and to reviewer #4 for their constructive suggestions towards validating the Kolmogorov–Smirnov (KS) filtering step. In response to the final reports of the two reviewers, we have performed a systematic differential expression (DE) analysis on the genes resulting from dropout filtering, using a design matrix that accounts for all the different condition pairs existing in our experimental design, namely phenotype, age group, time after injury, and cell type. As expected by reviewer #4, we see a substantial overlap between the significant genes picked up by the KS filtering step and the results of the DE analysis, when comparing the senescent and non-senescent phenotypes across conditions. Specifically, around 98% of the genes considered significant by the KS test are also significant according to the DE analysis, as implemented by the Python package PyDESeq2. This result is shown in the figure below, which is now included in the manuscript as Figure S2.

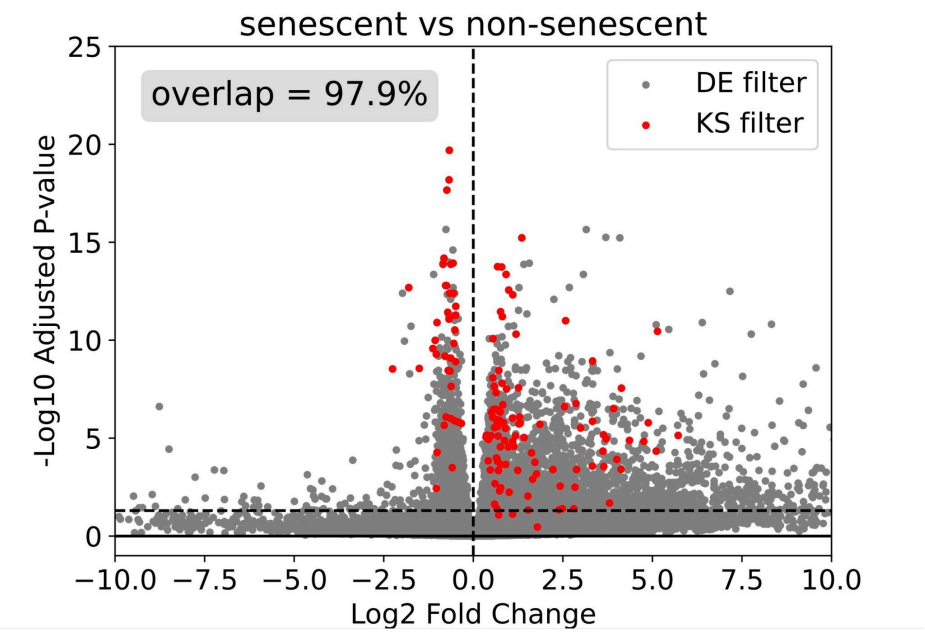

This figure shows the statistical significance vs fold change for all genes resulting from the dropout filtering (28,603 genes), highlighting in red those that were identified by the KS filtering approach. As can be seen in the figure, most of the KS-filtered genes are considered significant by the standard DE analysis as well.

Furthermore, we also used the DE analysis to compare the different condition pairs of our experimental dataset, with the aim of establishing the degree at which confounding by cell type,

age, or time point could influence our results. The figure below, included in this resubmission as Fig. S3, compares the DE statistical significance (measured by the logarithm of the adjusted p-value) of all the genes resulting from the dropout filtering, with that of the KS-filtered genes, for all pairwise combinations of conditions (including in the bottom row the three pairs of cell-type comparisons). The p-value distributions are normalized to unit area for a better comparison.

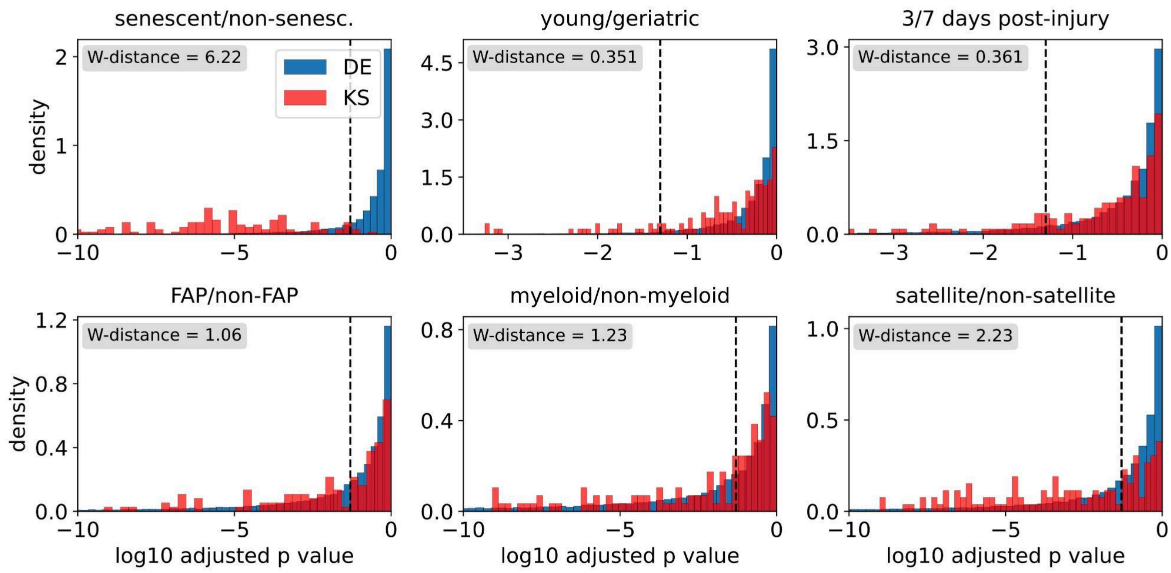

The results show that the KS-filtered genes (in red) are mostly significant (i.e. lie to the left of the significance threshold p=0.05 marked by the vertical dashed line) only for the senescence vs non-senescence comparison (top left panel). In contrast, for all other pairwise condition comparisons, the p-value distributions of the KS-filtered subset are similar to those of all the (dropout-filtered) genes. We quantified the distance between the distributions using the Wasserstein metric (grey-shadowed insets), which is between 3 and 20 times larger for the senescence vs non-senescence comparison than for the rest of the condition pairs. For the latter, most of the KS-filtered genes are below the significance level (to the right of the corresponding vertical dashed lines) of the DE analysis. This indicates that when comparing condition pairs other than the phenotype (senescent vs non-senescent), namely age group, time after injury, and cell type, the KS-filtered genes are basically a random sampling of all dropout-filtered genes. We thus conclude that confounding by cell type, age group, or time after injury is unlikely to influence the results shown in the manuscript.

We hope the analysis of this alternative approach, following the request of reviewer #3 and the specific suggestion of reviewer #4, further validates our pipeline and makes the paper adequate for publication in *PLoS Computational Biology*. This analysis is now mentioned in the main text of the paper (lines 154-167), and the two figures above are now shown as Figures S2 and S3 in the revised manuscript. The Python notebook used to perform this analysis has also been added to the paper's GitHub repository (https://github.com/dsb-lab/CellularSenescence).
