# Supplementary material for: Cell-type resolved transcriptional network analysis of in vivo cellular senescence following injury
Source: PLoS Comput Biol. 2026 Jun 22;22(6):e1014429. doi: 10.1371/journal.pcbi.1014429 (PMC13309044; doi:10.1371/journal.pcbi.1014429)
Supplement: Peer review history

# Peer review: Decision Letter 0

**DOI:** 10.1371/journal.pcbi.1014429.r001

**Authors:** Mark Alber, Jennifer Wilson

3 Sep 2025

PCOMPBIOL-D-25-01276

Cell-type resolved transcriptional networks reveal molecular hallmarks of cellular senescence

PLOS Computational Biology

Dear Dr. Garcia-Ojalvo,

Thank you for submitting your manuscript to PLOS Computational Biology. After careful consideration, we feel that it has merit but does not fully meet PLOS Computational Biology's publication criteria as it currently stands. Therefore, we invite you to submit a revised version of the manuscript that addresses the points raised during the review process. Especially, reviewers noted the necessity of citing other foundational work in the field and further differentiating your methodological contributions.

Please submit your revised manuscript within 60 days Nov 03 2025 11:59PM. If you will need more time than this to complete your revisions, please reply to this message or contact the journal office at ploscompbiol@plos.org. When you're ready to submit your revision, log on to https://www.editorialmanager.com/pcompbiol/ and select the 'Submissions Needing Revision' folder to locate your manuscript file.

Please include the following items when submitting your revised manuscript:

* A rebuttal letter that responds to each point raised by the editor and reviewer(s). You should upload this letter as a separate file labeled 'Response to Reviewers'. This file does not need to include responses to formatting updates and technical items listed in the 'Journal Requirements' section below.

* A marked-up copy of your manuscript that highlights changes made to the original version. You should upload this as a separate file labeled 'Revised Manuscript with Track Changes'.

* An unmarked version of your revised paper without tracked changes. You should upload this as a separate file labeled 'Manuscript'.

If you would like to make changes to your financial disclosure, competing interests statement, or data availability statement, please make these updates within the submission form at the time of resubmission. Guidelines for resubmitting your figure files are available below the reviewer comments at the end of this letter

We look forward to receiving your revised manuscript.

Kind regards,

Jennifer Wilson

Academic Editor

PLOS Computational Biology

Mark Alber

Section Editor

PLOS Computational Biology

**Journal Requirements:**

1) Please ensure that the CRediT author contributions listed for every co-author are completed accurately and in full.

At this stage, the following Authors/Authors require contributions: Alda Sabalic, Victoria Moiseeva, Oleg Deryagin, Andres Cisneros, Eusebio Perdiguero, Pura Munoz-Canoves, and Jordi Garcia-Ojalvo. Please ensure that the full contributions of each author are acknowledged in the "Add/Edit/Remove Authors" section of our submission form.

The list of CRediT author contributions may be found here: https://journals.plos.org/ploscompbiol/s/authorship#loc-author-contributions

2) We ask that a manuscript source file is provided at Revision. Please upload your manuscript file as a .doc, .docx, .rtf or .tex. If you are providing a .tex file, please upload it under the item type u2018LaTeX Source Fileu2019 and leave your .pdf version as the item type u2018Manuscriptu2019.

3) Please provide an Author Summary. This should appear in your manuscript between the Abstract (if applicable) and the Introduction, and should be 150-200 words long. The aim should be to make your findings accessible to a wide audience that includes both scientists and non-scientists. Sample summaries can be found on our website under Submission Guidelines:

https://journals.plos.org/ploscompbiol/s/submission-guidelines#loc-parts-of-a-submission

4) Your manuscript is missing the following sections: Results, and Discussion.  Please ensure all required sections are present and in the correct order. Make sure section heading levels are clearly indicated in the manuscript text, and limit sub-sections to 3 heading levels. An outline of the required sections can be consulted in our submission guidelines here:

https://journals.plos.org/ploscompbiol/s/submission-guidelines#loc-parts-of-a-submission

5) Please upload all main figures as separate Figure files in .tif or .eps format. For more information about how to convert and format your figure files please see our guidelines:

https://journals.plos.org/ploscompbiol/s/figures

6) Some material included in your submission may be copyrighted. According to PLOSu2019s copyright policy, authors who use figures or other material (e.g., graphics, clipart, maps) from another author or copyright holder must demonstrate or obtain permission to publish this material under the Creative Commons Attribution 4.0 International (CC BY 4.0) License used by PLOS journals. Please closely review the details of PLOSu2019s copyright requirements here: PLOS Licenses and Copyright. If you need to request permissions from a copyright holder, you may use PLOS's Copyright Content Permission form.

Please respond directly to this email and provide any known details concerning your material's license terms and permissions required for reuse, even if you have not yet obtained copyright permissions or are unsure of your material's copyright compatibility. Once you have responded and addressed all other outstanding technical requirements, you may resubmit your manuscript within Editorial Manager.

Potential Copyright Issues:

i) Figure 1. Please confirm whether you drew the images / clip-art within the figure panels by hand. If you did not draw the images, please provide (a) a link to the source of the images or icons and their license / terms of use; or (b) written permission from the copyright holder to publish the images or icons under our CC BY 4.0 license. Alternatively, you may replace the images with open source alternatives. See these open source resources you may use to replace images / clip-art:

- https://commons.wikimedia.org

- https://openclipart.org/.

7) Please amend your detailed Financial Disclosure statement. This is published with the article. It must therefore be completed in full sentences and contain the exact wording you wish to be published.

1) State the initials, alongside each funding source, of each author to receive each grant. For example: "This work was supported by the National Institutes of Health (####### to AM; ###### to CJ) and the National Science Foundation (###### to AM)."

2) State what role the funders took in the study. If the funders had no role in your study, please state: "The funders had no role in study design, data collection and analysis, decision to publish, or preparation of the manuscript."

3) If any authors received a salary from any of your funders, please state which authors and which funders.

8) Please ensure that the funders and grant numbers match between the Financial Disclosure field and the Funding Information tab in your submission form. Note that the funders must be provided in the same order in both places as well.

Note: If the reviewer comments include a recommendation to cite specific previously published works, please review and evaluate these publications to determine whether they are relevant and should be cited. There is no requirement to cite these works unless the editor has indicated otherwise.

**Reviewers' comments:**

Reviewer's Responses to Questions

Reviewer #1: In their ms, Sabalic and coworkers present an integrative analysis combining bulk RNA-seq from FACS-sorted muscle cells with network-based feature selection and community detection to identify transcriptional correlates of cellular senescence. They apply eigenvector centrality-based ranking and entropy-guided clustering and identify a minimal gene set of six genes (including Lncpint, Fabp3, Cd59a) that distinguishes senescent, non-senescent, and basal states across multiple cell types. They validated their key findings in an independent single-cell RNA-seq dataset from fibrotic liver tissue.

The study addresses a biologically significant and computationally challenging problem by identifying robust molecular markers for molecular characterization of cellular senescence. The methodology is clearly described, and the code is made available via GitHub. The validation by using independent scRNA-seq data enhances confidence in the biological relevance and generalizability of the results.

The manuscript is well structured and written with a concise message.

Major Comments

1. While the results are compelling and rather convincing, the ms would benefit from a more detailed comparison with between their ECFS and community detection approach with other methodologies like WGCNA, random forest feature selection or detailed PCA and ICA analyses. This would help the readers to contextualize the novelty and utility of their pipeline. This said, a more comprehensive description of the PCA analysis would be also interesting in terms of the explained variability of the different PCs, how the other PCs may discriminate the different cell states and if there is biological meaningful information in the PC loads/weights.

2. The biological interpretation how the gene candidates relate to senescence could be more extensive. Particularly, Cd59a would benefit from deeper discussion of potential mechanisms or prior evidence in senescence biology. Furthermore, some more detailed discussion on the limitations of the study would allow to put the results in a broader biological context. While the confirmation in liver data is interesting, the potential context could be discussed further.

3. Some more clarification on the experimental set-up is needed. Were some experiments performed for this study or was it all done in relation to ref 23? Accordingly to the statement “There are no primary data in the paper.“ no experiments were performed, but then the FACS sorting description in Sec. 8.1 is confusing. If experiments were done for this study, the ethics should be also specified.

Minor comments

- Page 2: replace “-“ with “,” or adda space in “… between their elements –which in the context of this investigation”.

- Page 7: use lower case “s” in “is explained in the next Section.”.

- Page 9: Something is missing in the sentence “Similar to the mixing of basal and non-senescent cells, this might be because both of these cell states are extracted from the injured leg.”?

- Page 12: Please clarify the sentence “It is worth noting that although the cell types used for validation originated from an entirely distinct tissue from those in our analysis, we could use our results to find the senescent cells that overlapped with our dataset, indicating that our conclusions for myeloid cells might be generalizable.”

- It would be nice to have a README in the github repository giving the context of the repository and maybe also mention the corresponding python version. For me it worked at least with python 3.9.17.

- It would be interesting to see the distribution of the ECSF sores to have a better intuition on the gene selection.

- What is the reasoning for 1.5 as a threshold in splitting the dTomato population in Figure 7?

Besides these comments, the manuscript could be a very valuable contribution to PLoS Computational Biology when the points above are addressed accordingly.

Reviewer #2: The authors have used cell-type resolved bulk RNA-seq with eigenvector centrality analysis and community detection (Louvain) to compress senescence vs. non-senescence transcriptomes into a small discriminative set pf six genes that separates cell states across FAPs, myeloid cells, and satellite cells during muscle regeneration after injury. Additional analysis is used to quantify the robustness of the separation analysis. As far as I know these approaches are sound and original and the findings. While the analysis highlights some potentially novel candidate markers of senescence in the context of skeletal muscle regeneration and their transcriptional regulatory networks, I am less certain about the originality and significance of these findings given that several major studies in the field appear not to have been cited.

• Ogrodnik M, Carlos Acosta J, Adams PD, d'Adda di Fagagna F, Baker DJ, Bishop CL, Chandra T, Collado M, Gil J, Gorgoulis V, Gruber F, Hara E, Jansen-Dürr P, Jurk D, Khosla S, Kirkland JL, Krizhanovsky V, Minamino T, Niedernhofer LJ, Passos JF, Ring NAR, Redl H, Robbins PD, Rodier F, Scharffetter-Kochanek K, Sedivy JM, Sikora E, Witwer K, von Zglinicki T, Yun MH, Grillari J, Demaria M. Guidelines for minimal information on cellular senescence experimentation in vivo. Cell 2024 Aug 8;187(16):4150-4175. doi: 10.1016/j.cell.2024.05.059. PMID: 39121846; PMCID: PMC11790242.

This paper describes minimum information for cellular senescence experimentation in vivo that the authors should acknowledge and confirm that they followed.

• Suryadevara V, Hudgins AD, Rajesh A, Pappalardo A, Karpova A, Dey AK, Hertzel A, Agudelo A, Rocha A, Soygur B, Schilling B, Carver CM, Aguayo-Mazzucato C, Baker DJ, Bernlohr DA, Jurk D, Mangarova DB, Quardokus EM, Enninga EAL, Schmidt EL, Chen F, Duncan FE, Cambuli F, Kaur G, Kuchel GA, Lee G, Daldrup-Link HE, Martini H, Phatnani H, Al-Naggar IM, Rahman I, Nie J, Passos JF, Silverstein JC, Campisi J, Wang J, Iwasaki K, Barbosa K, Metis K, Nernekli K, Niedernhofer LJ, Ding L, Wang L, Adams LC, Ruiyang L, Doolittle ML, Teneche MG, Schafer MJ, Xu M, Hajipour M, Boroumand M, Basisty N, Sloan N, Slavov N, Kuksenko O, Robson P, Gomez PT, Vasilikos P, Adams PD, Carapeto P, Zhu Q, Ramasamy R, Perez-Lorenzo R, Fan R, Dong R, Montgomery RR, Shaikh S, Vickovic S, Yin S, Kang S, Suvakov S, Khosla S, Garovic VD, Menon V, Xu Y, Song Y, Suh Y, Dou Z, Neretti N. SenNet recommendations for detecting senescent cells in different tissues. Nat Rev Mol Cell Biol. 2024 Dec;25(12):1001-1023. doi: 10.1038/s41580-024-00738-8. Epub 2024 Jun 3. PMID: 38831121; PMCID: PMC11578798.

This article includes recommendations for detecting senescent cells in based on literature reported senescence markers in 14 tissues in mice and humans. It seems highly relevant but is not cited or discussed.

• Li Y, Li C, Zhou Q, Liu X, Qiao Y, Xie T, Sun H, Ong MT, Wang H. Multiomics and cellular senescence profiling of aging human skeletal muscle uncovers Maraviroc as a senotherapeutic approach for sarcopenia. Nat Commun. 2025 Jul 5;16(1):6207. doi: 10.1038/s41467-025-61403-y. PMID: 40617829; PMCID: PMC12228793.

This paper reports the first senescence atlas profiling senescence in human skeletal muscle.

• Walter LD, Orton JL, Ntekas I, Fong EHH, Maymi VI, Rudd BD, De Vlaminck I, Elisseeff JH, Cosgrove BD. Transcriptomic analysis of skeletal muscle regeneration across mouse lifespan identifies altered stem cell states. Nat Aging. 2024 Dec;4(12):1862-1881. doi: 10.1038/s43587-024-00756-3. Epub 2024 Nov 22. PMID: 39578558; PMCID: PMC11645289.

Although focused more specifically on stem cell states, this paper identifies molecular markers of age-related renerative potential.

In summary, the manuscript reports a compact, network-derived gene set that separates senescent from non-senescent states across muscle cell types in regeneration, but it ignores major recent multiomic studies in the field. This casts some doubt on the extent to which the authors have really identified novel “hallmarks” of skeletal muscle senescence.

Reviewer #3: Sabalic et al. report novel targets of cellular senescence in a skeletal muscle injury model. The paper primarily focuses on the development of computational methods to identify senescent cells within a mixed cell population, rather than presenting new biological findings. However, a general definition of senescent cells, including well-known biomarkers, should be provided. Additionally, the use of cells identified as senescent in aged humans or model organisms—rather than those resulting from artificially-induced muscle injury—may be necessary to justify the methodological approach.

Since this study is predominantly based on a chemically induced muscle injury model, both the overall scope and the title should more accurately reflect this focus instead of adopting a broadly generalized view. This consideration should also be integrated into the Introduction section, given that the model system was previously reported by the co-authors (Moiseeva et al., Nature 2023, doi: 10.1038/s41586-022-05535-x). Because the earlier paper employed a very similar mouse muscle injury model for both bulk and single-cell RNA sequencing, it is important to highlight the biological and methodological differences and the novel aspects of this current study.

Similar bioinformatic approaches using eigenvector centrality have been published for RNA-seq network community analysis by multiple groups, although none have specifically focused on senescence or muscle injury (Pashaei et al., NAR 2025, doi: 10.1093/NAR/GKAF609; Sarmah and Bhattacharyya, J Biosci 2024, doi: 10.1007/s12038-024-00437-8; Wei et al., LSA 2023, doi: 10.26508/LSA.202302253; Liu et al., iScience 2018, doi: 10.1016/J.ISCI.2018.08.002). If the authors’ methodology is primarily computational, they need to clearly articulate what makes their approach novel or more advantageous compared to these existing methods.

Given that the utility of the current approach for identifying novel and generalizable senescence markers is considered, the analysis should be extended to more published datasets, coupled with biological validation. Currently, the manuscript lacks clarity throughout; a thorough revision is recommended to emphasize the novel methodological and biological contributions.

Other Issues:

1. The first paragraph of the Introduction should adopt a broader perspective, as cellular aging is inherently heterogeneous. For example, some senescence pathways are not triggered by molecular damage, whereas DNA damage remains an important cause—either directly or indirectly—of senescence. This may be a writing issue, as heterogeneity is acknowledged later in the manuscript.

2. In relation to the above, it would be more effective to include an overview of the muscle injury model within the context of senescence research, given that this is the primary focus of the study.

3. While citing many review articles can be helpful, the authors should incorporate more primary research articles where relevant to strengthen their arguments.

4. The Experimental Data section should specify the sources of key materials, such as cardiotoxin and the mouse strain used, to ensure reproducibility. This basic information is currently lacking.

5. The statement, “We focused on three different cell types because of their crucial role in muscle tissue regeneration—satellite cells (muscle stem cells), fibroadipogenic progenitor (FAP) cells, and myeloid cells”—raises questions about their specific roles and the rationale behind selecting these cell types. Clarification is needed, particularly since the focus on these cells may be specific to this experimental model rather than applicable to cellular senescence broadly.

6. The manuscript should specify which gene database was used for RNA-seq analysis (e.g., GENCODE, REFSEQ, or others). Although the authors mention a total of 46,078 genes, the specific database and version used should be clearly indicated, as gene counts can vary between releases.

7. Providing clearer information about the genes identified in Table 1 and Figure 3 would be helpful. Notably, the color-coding legend in Figure 3 appears to use the same blue for both cell cycle and apoptosis resistance, which could be confusing. Furthermore, since transcription factors are depicted hierarchically, a label like “gene expression” is too broad; the database used for functional annotations should be cited. For example, there appears to be no match for Elf1 in mouse or human KEGG pathways, despite the authors’ statement. Additionally, the phrase “The pathways shown are related to established senescence” in the figure legend would benefit from specific citations or source references.

8. In Figure 7, the graph appears to represent a histogram, but it is unclear what scale is being used. Since tdTomato expression is intended to report p16 levels, clarification is needed. The cited Cell Metabolism paper describes tissues in terms of p16^high^ or p16^low^, reflecting their differing distributions of p16. The authors should clearly explain what they did in the current study to reassess the previously reported p16 data (Figures 7 and 8).

9. Regarding the computational implementations, did the authors use the MATLAB code provided in the cited paper on the eigenvector centrality ranking algorithm (Roffo and Melzi, 2016)? The mathematical formulations in Sections 8.2–8.3 are already presented in that paper, unless the authors introduced modifications. The current manuscript provides no information on how the method was actually implemented—whether as a computational package or as executable resources—which is a significant omission.

**********

**Have the authors made all data and (if applicable) computational code underlying the findings in their manuscript fully available?**

The PLOS Data policy requires authors to make all data and code underlying the findings described in their manuscript fully available without restriction, with rare exception (please refer to the Data Availability Statement in the manuscript PDF file). The data and code should be provided as part of the manuscript or its supporting information, or deposited to a public repository. For example, in addition to summary statistics, the data points behind means, medians and variance measures should be available. If there are restrictions on publicly sharing data or code —e.g. participant privacy or use of data from a third party—those must be specified.

Reviewer #1: Yes

Reviewer #2: Yes

Reviewer #3: **No:** Written in the comments

**********

PLOS authors have the option to publish the peer review history of their article (what does this mean?). If published, this will include your full peer review and any attached files.

If you choose “no”, your identity will remain anonymous but your review may still be made public.

**Do you want your identity to be public for this peer review?**  For information about this choice, including consent withdrawal, please see our Privacy Policy.

Reviewer #1: No

Reviewer #2: No

Reviewer #3: No

[NOTE: If reviewer comments were submitted as an attachment file, they will be attached to this email and accessible via the submission site. Please log into your account, locate the manuscript record, and check for the action link "View Attachments". If this link does not appear, there are no attachment files.]

**Figure resubmission:**

While revising your submission, we strongly recommend that you use PLOS’s NAAS tool (https://ngplosjournals.pagemajik.ai/artanalysis) to test your figure files. NAAS can convert your figure files to the TIFF file type and meet basic requirements (such as print size, resolution), or provide you with a report on issues that do not meet our requirements and that NAAS cannot fix.

After uploading your figures to PLOS’s NAAS tool - https://ngplosjournals.pagemajik.ai/artanalysis, NAAS will process the files provided and display the results in the "Uploaded Files" section of the page as the processing is complete. If the uploaded figures meet our requirements (or NAAS is able to fix the files to meet our requirements), the figure will be marked as "fixed" above. If NAAS is unable to fix the files, a red "failed" label will appear above. When NAAS has confirmed that the figure files meet our requirements, please download the file via the download option, and include these NAAS processed figure files when submitting your revised manuscript.

**Reproducibility:**

To enhance the reproducibility of your results, we recommend that authors of applicable studies deposit laboratory protocols in protocols.io, where a protocol can be assigned its own identifier (DOI) such that it can be cited independently in the future. Additionally, PLOS ONE offers an option to publish peer-reviewed clinical study protocols. Read more information on sharing protocols at https://plos.org/protocols?utm_medium=editorial-email&utm_source=authorletters&utm_campaign=protocols

# Author comment: Author response to Decision Letter 1

**DOI:** 10.1371/journal.pcbi.1014429.r002

17 Dec 2025

Attachment Submitted filename: senescence_response_to_reviewers.pdf

# Peer review: Decision Letter 1

**DOI:** 10.1371/journal.pcbi.1014429.r003

**Authors:** Mark Alber, Jennifer Wilson, Christoph Kaleta

26 Jan 2026

PCOMPBIOL-D-25-01276R1

Cell-type resolved transcriptional network analysis of in vivo cellular senescence following injury

PLOS Computational Biology

Dear Dr. Garcia-Ojalvo,

Thank you for submitting your manuscript to PLOS Computational Biology. After careful consideration, we feel that it has merit but does not fully meet PLOS Computational Biology's publication criteria as it currently stands. Therefore, we invite you to submit a revised version of the manuscript that addresses the points raised during the review process.

Please submit your revised manuscript by Mar 28 2026 11:59PM. If you will need more time than this to complete your revisions, please reply to this message or contact the journal office at ploscompbiol@plos.org. When you're ready to submit your revision, log on to https://www.editorialmanager.com/pcompbiol/ and select the 'Submissions Needing Revision' folder to locate your manuscript file.

Please include the following items when submitting your revised manuscript:

* A letter that responds to each point raised by the editor and reviewer(s). You should upload this letter as a separate file labeled 'Response to Reviewers'. This file does not need to include responses to formatting updates and technical items listed in the 'Journal Requirements' section below.

* A marked-up copy of your manuscript that highlights changes made to the original version. You should upload this as a separate file labeled 'Revised Manuscript with Track Changes'.

* An unmarked version of your revised paper without tracked changes. You should upload this as a separate file labeled 'Manuscript'.

If you would like to make changes to your financial disclosure, competing interests statement, or data availability statement, please make these updates within the submission form at the time of resubmission. Guidelines for resubmitting your figure files are available below the reviewer comments at the end of this letter.

We look forward to receiving your revised manuscript.

Kind regards,

Christoph Kaleta

Section Editor

PLOS Computational Biology

Mark Alber

Section Editor

PLOS Computational Biology

**Journal Requirements:**

If the reviewer comments include a recommendation to cite specific previously published works, please review and evaluate these publications to determine whether they are relevant and should be cited. There is no requirement to cite these works unless the editor has indicated otherwise.

**Reviewers' comments:**

Reviewer's Responses to Questions

**Comments to the Authors:**

**Please note here if the review is uploaded as an attachment.**

Reviewer #2: The authors have satisfactorily addressed my review comments

Reviewer #3: The revised manuscript by Sabalic et al. now includes many essential lines of information that was lacking in the original submission. As a result, its readability has been improved substantially for broad audience.

Remaining Issue:

A minor point first but in Figure 3 Significant filtering -- it appears the same as the 'discriminating filtering' shown in Figure 1. Filtering (and RNA-seq in general) is using statistical criteria (in this case the KS test). For this, using one term will reduce confusion.

More importantly, however, I must question whether the Kolmogorov-Smirnov test is really appropriate to compare the means of gene 'i' expression between sample groups with small sample size. In a total of the 36 samples without counting replicates, the sample size of each group could be around 10-20 (looks like 12 from the right panel of Fig 3B), considered too small below the generalized effective size range of 50 and 1000 for KS test. Although the authors applied Bonferroni correction, it tries to control overall Type I errors but it does not mitigate this issue. Therefore, it is advisable to find an alternative that tolerates small sample sizes better than KS test.

In the same token, I wonder if a negative binomial method may simply suffice without separating the two filtering steps during bulk RNA-seq analysis as the authors used in Ref.20. Simply, the use of ranking by the degree of differential expression available in the DEG list generated previously by EdgeR, may work similarly without raising the sample size issue above. Have the authors already tried it? Since the authors' main point of this manuscript is about application of the eigenvector centrality feature selection algorithm developed by another group earlier for network community analysis, the goal of RNA-seq analysis may be relatively peripheral. However, it is important to find a confidence in selected approach.

By choosing a different test appropriate for small sample sizes in the second filtering step (or whole in RNA-seq data analysis), I believe that the manuscript requires a revision. Depending on the outcome, results and discussion (as well as methods) may also need adjustments.

**********

**Have the authors made all data and (if applicable) computational code underlying the findings in their manuscript fully available?**

The PLOS Data policy requires authors to make all data and code underlying the findings described in their manuscript fully available without restriction, with rare exception (please refer to the Data Availability Statement in the manuscript PDF file). The data and code should be provided as part of the manuscript or its supporting information, or deposited to a public repository. For example, in addition to summary statistics, the data points behind means, medians and variance measures should be available. If there are restrictions on publicly sharing data or code —e.g. participant privacy or use of data from a third party—those must be specified.

Reviewer #2: Yes

Reviewer #3: Yes

**********

PLOS authors have the option to publish the peer review history of their article (what does this mean?). If published, this will include your full peer review and any attached files.

If you choose “no”, your identity will remain anonymous but your review may still be made public.

**Do you want your identity to be public for this peer review?**  For information about this choice, including consent withdrawal, please see our Privacy Policy.

Reviewer #2: No

Reviewer #3: No

**Figure resubmission:**

-->While revising your submission, we strongly recommend that you use PLOS’s NAAS tool (https://ngplosjournals.pagemajik.ai/artanalysis) to test your figure files. NAAS can convert your figure files to the TIFF file type and meet basic requirements (such as print size, resolution), or provide you with a report on issues that do not meet our requirements and that NAAS cannot fix.

After uploading your figures to PLOS’s NAAS tool - https://ngplosjournals.pagemajik.ai/artanalysis, NAAS will process the files provided and display the results in the "Uploaded Files" section of the page as the processing is complete. If the uploaded figures meet our requirements (or NAAS is able to fix the files to meet our requirements), the figure will be marked as "fixed" above. If NAAS is unable to fix the files, a red "failed" label will appear above. When NAAS has confirmed that the figure files meet our requirements, please download the file via the download option, and include these NAAS processed figure files when submitting your revised manuscript.

**Reproducibility:**

To enhance the reproducibility of your results, we recommend that authors of applicable studies deposit laboratory protocols in protocols.io, where a protocol can be assigned its own identifier (DOI) such that it can be cited independently in the future. Additionally, PLOS ONE offers an option to publish peer-reviewed clinical study protocols. Read more information on sharing protocols at https://plos.org/protocols?utm_medium=editorial-email&utm_source=authorletters&utm_campaign=protocols

# Author comment: Author response to Decision Letter 2

**DOI:** 10.1371/journal.pcbi.1014429.r004

26 Feb 2026

Attachment Submitted filename: senescence_response_to_reviewers_2.pdf

# Peer review: Decision Letter 2

**DOI:** 10.1371/journal.pcbi.1014429.r005

**Authors:** Mark Alber, Jennifer Wilson, Christoph Kaleta, Christoph Kaleta

8 Apr 2026

PCOMPBIOL-D-25-01276R2

Cell-type resolved transcriptional network analysis of in vivo cellular senescence following injury

PLOS Computational Biology

Dear Dr. Garcia-Ojalvo,

Thank you for submitting your manuscript to PLOS Computational Biology. After careful consideration, we feel that it has merit but does not fully meet PLOS Computational Biology's publication criteria as it currently stands. Following the assessment of one reviewer that their statistical concerns may not have been fully addressed, another reviewer was invited to consider those concerns. Based on their assessment, I'd like to ask you to use their advice how to remedy those concerns.

Please submit your revised manuscript by Jun 08 2026 11:59PM. If you will need more time than this to complete your revisions, please reply to this message or contact the journal office at ploscompbiol@plos.org. When you're ready to submit your revision, log on to https://www.editorialmanager.com/pcompbiol/ and select the 'Submissions Needing Revision' folder to locate your manuscript file.

Please include the following items when submitting your revised manuscript:

* A letter that responds to each point raised by the editor and reviewer(s). You should upload this letter as a separate file labeled 'Response to Reviewers'. This file does not need to include responses to formatting updates and technical items listed in the 'Journal Requirements' section below.

* A marked-up copy of your manuscript that highlights changes made to the original version. You should upload this as a separate file labeled 'Revised Manuscript with Track Changes'.

* An unmarked version of your revised paper without tracked changes. You should upload this as a separate file labeled 'Manuscript'.

If you would like to make changes to your financial disclosure, competing interests statement, or data availability statement, please make these updates within the submission form at the time of resubmission. Guidelines for resubmitting your figure files are available below the reviewer comments at the end of this letter.

We look forward to receiving your revised manuscript.

Kind regards,

Christoph Kaleta

Section Editor

PLOS Computational Biology

Mark Alber

Section Editor

PLOS Computational Biology

**Journal Requirements:**

If the reviewer comments include a recommendation to cite specific previously published works, please review and evaluate these publications to determine whether they are relevant and should be cited. There is no requirement to cite these works unless the editor has indicated otherwise.

**Reviewers' comments:**

Reviewer's Responses to Questions

**Comments to the Authors:**

**Please note here if the review is uploaded as an attachment.**

Reviewer #3: The authors have revised the manuscript in response to the previous comments regarding the applicability of the Kolmogorov–Smirnov (KS) test. In the revised version, the authors state that the KS test was conducted using 108 samples (36 groups × 3 replicates). Based on this clarification, however, I remain concerned about the validity of the test, as the current description appears to overlook the distinction between the intra-group distribution (across replicates) and the inter-group distribution (between groups).

Because this test is used as a key gene-filtering step that feeds directly into the subsequent network construction, the statistical validity of this step is important for evaluating the reliability of the downstream results.

If the primary goal of this step is to reduce or filter the number of genes, several alternative approaches could be considered, such as applying a stringent p-value threshold, fold-change filtering, variance-based filtering, or potentially a mixed-effects model. The way the KS test is applied remains somewhat unclear from a statistical perspective, and further clarification or reconsideration of this step would be appreciated.

Reviewer #4: I appreciate the authors’ clarification of the application of the Kolmogorov-Smirnov test. The modified figures and updated pipeline description improve the original ambiguity in the manuscript. It is acknowledged that the sample sizes are larger than what was initially inferred from the previous version of the figures.

However, my primary concern is not yet fully addressed. Considering the new information about sample composition, the issue seems to extend beyond sample size to the independence assumption underlying the KS test. The 36 senescent observations are not independent samples from a single "senescent" distribution. Rather, they seem to comprise a structured combination of 3 cell types, 2 age groups, 2 time points, and 3 biological replicates. This structure also applies to the 72 non-senescent and basal observations.

This pooling approach may cause the test to identify genes that differ systematically between cell types, age groups, or time points, rather than specifically between senescent and non-senescent states. For example, if the composition of cell types or age groups differs even just slightly between the senescent and combined non-senescent/basal groups, the KS test would detect this compositional difference instead of a true senescence effect. While the Bonferroni correction controls the familywise error rate under valid test assumptions, it does not resolve any violations of those assumptions.

I’d like to emphasize that I do not consider this issue to be fatal to the manuscript. In this case, the KS test serves as an intermediate filtering step, and downstream eigenvector centrality and community-detection analyses provide further refinement. The final set of genes is biologically coherent, which supports confidence in the overall pipeline. However, the statistical methodology at this stage should be defensible on its own merits, especially in a manuscript proposing a computational workflow for wider use.

I therefore would appreciate it if the authors could undertake one of the following actions:

(1) Conduct a sensitivity analysis comparing the genes identified by the KS test to those identified by either a standard differential expression framework (such as edgeR or DESeq2) using an appropriate design matrix that accounts for cell type, age group, and time point, or at the very least a statistical test that is more robust to small sample sizes and dependent conditions. If the overlap is substantial, this would demonstrate that the downstream results are robust to the choice of filtering method and would wholly resolve my concern.

(2) Alternatively, if the authors prefer to maintain the KS test, they should provide an explicit discussion acknowledging that the test does not account for the nested experimental structure, along with an explanation for why this is acceptable in the context of a coarse pre-filtering step. This should include, at a minimum, a brief argument for why confounding by cell type, age, or time point is unlikely to influence the results.

I consider either of these requests minor in scope and do not anticipate that they would require extensive changes to the results or conclusions. My expectation is that option (1) would likely confirm the robustness of the existing findings and thereby significantly strengthen the manuscript.

**********

**Have the authors made all data and (if applicable) computational code underlying the findings in their manuscript fully available?**

The PLOS Data policy requires authors to make all data and code underlying the findings described in their manuscript fully available without restriction, with rare exception (please refer to the Data Availability Statement in the manuscript PDF file). The data and code should be provided as part of the manuscript or its supporting information, or deposited to a public repository. For example, in addition to summary statistics, the data points behind means, medians and variance measures should be available. If there are restrictions on publicly sharing data or code —e.g. participant privacy or use of data from a third party—those must be specified.

Reviewer #3: Yes

Reviewer #4: None

**********

PLOS authors have the option to publish the peer review history of their article (what does this mean?). If published, this will include your full peer review and any attached files.

If you choose “no”, your identity will remain anonymous but your review may still be made public.

**Do you want your identity to be public for this peer review?**  For information about this choice, including consent withdrawal, please see our Privacy Policy.

Reviewer #3: No

Reviewer #4: No

**Figure resubmission:**

-->While revising your submission, we strongly recommend that you use PLOS’s NAAS tool (https://ngplosjournals.pagemajik.ai/artanalysis) to test your figure files. NAAS can convert your figure files to the TIFF file type and meet basic requirements (such as print size, resolution), or provide you with a report on issues that do not meet our requirements and that NAAS cannot fix.

After uploading your figures to PLOS’s NAAS tool - https://ngplosjournals.pagemajik.ai/artanalysis, NAAS will process the files provided and display the results in the "Uploaded Files" section of the page as the processing is complete. If the uploaded figures meet our requirements (or NAAS is able to fix the files to meet our requirements), the figure will be marked as "fixed" above. If NAAS is unable to fix the files, a red "failed" label will appear above. When NAAS has confirmed that the figure files meet our requirements, please download the file via the download option, and include these NAAS processed figure files when submitting your revised manuscript.

**Reproducibility:**

To enhance the reproducibility of your results, we recommend that authors of applicable studies deposit laboratory protocols in protocols.io, where a protocol can be assigned its own identifier (DOI) such that it can be cited independently in the future. Additionally, PLOS ONE offers an option to publish peer-reviewed clinical study protocols. Read more information on sharing protocols at https://plos.org/protocols?utm_medium=editorial-email&utm_source=authorletters&utm_campaign=protocols

# Author comment: Author response to Decision Letter 3

**DOI:** 10.1371/journal.pcbi.1014429.r006

8 Jun 2026

Attachment Submitted filename: senescence_response_to_reviewers_3.pdf

# Peer review: Decision Letter 3

**DOI:** 10.1371/journal.pcbi.1014429.r007

**Authors:** Mark Alber, Jennifer Wilson, Christoph Kaleta, Christoph Kaleta, Christoph Kaleta

10 Jun 2026

Dear Dr. Garcia-Ojalvo,

We are pleased to inform you that your manuscript 'Cell-type resolved transcriptional network analysis of in vivo cellular senescence following injury' has been provisionally accepted for publication in PLOS Computational Biology.

Before your manuscript can be formally accepted you will need to complete some formatting changes, which you will receive in a follow up email. A member of our team will be in touch with a set of requests.

Please note that your manuscript will not be scheduled for publication until you have made the required changes, so a swift response is appreciated.

IMPORTANT: The editorial review process is now complete. PLOS will only permit corrections to spelling, formatting or significant scientific errors from this point onwards. Requests for major changes, or any which affect the scientific understanding of your work, will cause delays to the publication date of your manuscript.

Should you, your institution's press office or the journal office choose to press release your paper, you will automatically be opted out of early publication. We ask that you notify us now if you or your institution is planning to press release the article. All press must be co-ordinated with PLOS.

Thank you again for supporting Open Access publishing; we are looking forward to publishing your work in PLOS Computational Biology.

Best regards,

Christoph Kaleta

Section Editor

PLOS Computational Biology

Mark Alber

Section Editor

PLOS Computational Biology

***********************************************************

Reviewer's Responses to Questions

**Comments to the Authors:**

**Please note here if the review is uploaded as an attachment.**

Reviewer #4: I would like to thank the authors for the time and effort they invested in this well-structured review. The team not only performed the requested sensitivity analysis but also went beyond that to address the confounding issue in figure S3. I follow their reasoning regarding the results of this new analysis and agree with their conclusions. My previous concerns have now been fully addressed and resolved. In my opinion, the paper has been strengthened more than sufficiently, and I see no further issues that would oppose publication.

**********

**Have the authors made all data and (if applicable) computational code underlying the findings in their manuscript fully available?**

The PLOS Data policy requires authors to make all data and code underlying the findings described in their manuscript fully available without restriction, with rare exception (please refer to the Data Availability Statement in the manuscript PDF file). The data and code should be provided as part of the manuscript or its supporting information, or deposited to a public repository. For example, in addition to summary statistics, the data points behind means, medians and variance measures should be available. If there are restrictions on publicly sharing data or code —e.g. participant privacy or use of data from a third party—those must be specified.

Reviewer #4: Yes

**********

PLOS authors have the option to publish the peer review history of their article (what does this mean?). If published, this will include your full peer review and any attached files.

If you choose “no”, your identity will remain anonymous but your review may still be made public.

**Do you want your identity to be public for this peer review?**  For information about this choice, including consent withdrawal, please see our Privacy Policy.

Reviewer #4: **Yes:** Kristina Schlicht

# Editor report: Acceptance letter

**DOI:** 10.1371/journal.pcbi.1014429.r008

**Authors:** Mark Alber, Jennifer Wilson, Christoph Kaleta, Christoph Kaleta, Christoph Kaleta

PCOMPBIOL-D-25-01276R3

Cell-type resolved transcriptional network analysis of in vivo cellular senescence following injury

Dear Dr Garcia-Ojalvo,

I am pleased to inform you that your manuscript has been formally accepted for publication in PLOS Computational Biology. Your manuscript is now with our production department and you will be notified of the publication date in due course.

The corresponding author will soon be receiving a typeset proof for review, to ensure errors have not been introduced during production. Please review the PDF proof of your manuscript carefully, as this is the last chance to correct any errors. Please note that major changes, or those which affect the scientific understanding of the work, will likely cause delays to the publication date of your manuscript.

Soon after your final files are uploaded, unless you have opted out, the early version of your manuscript will be published online. The date of the early version will be your article's publication date. The final article will be published to the same URL, and all versions of the paper will be accessible to readers.

For Research, Software, and Methods articles, you will receive an invoice from PLOS for your publication fee after your manuscript has reached the completed accept phase. If you receive an email requesting payment before acceptance or for any other service, this may be a phishing scheme. Learn how to identify phishing emails and protect your accounts at https://explore.plos.org/phishing.

Thank you again for supporting PLOS Computational Biology and open-access publishing. We are looking forward to publishing your work!

With kind regards,

Zsofia Freund

PLOS Computational Biology | Carlyle House, Carlyle Road, Cambridge CB4 3DN | United Kingdom ploscompbiol@plos.org | Phone +44 (0) 1223-442824 | ploscompbiol.org | @PLOSCompBiol
